# The prognostic value of the neutrophil-percentage-to-albumin ratio for all-cause and cardiovascular mortality in chronic kidney disease stages G3a to G5: insights from NHANES 2003–2018

**DOI:** 10.1080/0886022X.2025.2495861

**Published:** 2025-05-07

**Authors:** Jialing Rao, Yuanqing Li, Xiaohao Zhang, Wenbo Zhao, Yanru Chen, Jun Zhang, Hui Peng, Zengchun Ye

**Affiliations:** Department of Nephrology, The Third Affiliated Hospital of Sun Yat-Sen University, Guangzhou, China

**Keywords:** Neutrophil-percentage-to-albumin ratio, chronic kidney disease, all-cause mortality, cardiovascular disease mortality, NHANES

## Abstract

**Background:**

Patients with chronic kidney disease (CKD) stages G3a to G5 frequently experience heightened systemic inflammation and nutritional loss. Identifying laboratory-accessible, cost-effective markers that can effectively predict the prognosis of CKD stages G3a to G5 is crucial.

**Methods:**

This prospective cohort study included 3,331 patients with CKD stages G3a to G5 who participated in the National Health and Nutrition Examination Survey (NHANES) from 2003 to 2018. Multivariable adjusted Cox proportional hazards regression models and restricted cubic spline analyses were used to assess the associations of neutrophil percentage-to-albumin ratio (NPAR) levels with all-cause mortality, CVD, and non-CVD mortality.

**Results:**

The cohort study encompassed data from 3,331 participants for analysis. Nonlinear J-shaped associations were observed between NPAR levels and the risk of all-cause, CVD, and non-CVD mortality in patients with CKD stages G3a to G5. High levels NPAR exhibited a significantly elevated risk of both all-cause and CVD mortality in the fully adjusted model. The respective hazard ratios (HRs) for all-cause mortality were 1.23 [95% confidence interval (CI), 1.05–1.44], and for CVD mortality, 1.513 (95% CI, 1.131–2.024).

**Conclusions:**

Elevated NPAR can predict both all-cause and CVD deaths in advanced CKD patients. Individuals with high NPAR levels face an elevated risk of mortality and exhibit a decreased survival rate in the context of CKD. This finding offers evidence supporting the timely evaluation and intervention for inflammation and nutritional status in individuals with CKD stages G3a to G5.

## Introduction

Chronic kidney disease (CKD) is a global health issue. According to the third iteration of International Society of Nephrology (ISN) Global Kidney Health Atlas (ISN-GKHA) [1], the median prevalence of CKD worldwide is 9.5%. The median percentage of global Disability-Adjusted Life Years (DALYs) attributable to CKD is 1.5%, and the median mortality rate for global CKD is 2.4%. Chronic kidney disease imposes a heavy burden on public health, including treatment costs and socio-economic factors. Kidney Disease: Improving Global Outcomes (KDIGO) defines a GFR <60 mL/min/1.73 m^2^ for at least 3 months as GFR categories G3a–G5 [2]. A series of complications, such as anemia, cardiovascular and cerebrovascular events, mineral and bone disorders, malnutrition, and metabolic disturbances, begin to manifest when renal function declines below 60 mL/min/1.73 m^2^. These complications may accelerate progression, potentially leading to further kidney function decline and increased risk of mortality. Consequently, the evaluation and prediction of outcomes in patients with CKD stages G3a to G5 are of paramount importance.

Patients with CKD frequently experience heightened systemic inflammation and nutritional loss [3]. CKD fosters an inflammatory environment through various mechanisms, including oxidative stress, infections, dyslipidemia, malnutrition, and impaired clearance of inflammatory mediators. Concurrently, the increase in inflammatory factors contributes to the exacerbation of kidney damage [4]. In patients with CKD, inflammatory markers such as interleukin 1 (IL-1,elevated in dialysis patients), fibrinogen, and TNF-α are independent predictors of CKD progression [5]. Research has indicated that the onset of CKD is associated with heightened levels of metabolites from the kynurenine pathway (KP), which is the primary route for the breakdown of tryptophan (Trp). Elevated KP metabolites and an increased kynurenine: tryptophan ratio is linked to higher levels of inflammatory markers in individuals with CKD [6]. Epidermal growth factor (EGF), α-1 microglobulin, kidney injury molecule-1 (KIM1), and monocyte chemoattractant protein-1 (MCP-1) were associated with an elevated risk of CKD progression [7]. Chronic inflammation and malnutrition significantly contribute to both overall and cardiovascular (CVD) mortality among patients with CKD [8]. The inflammatory markers previously mentioned are generally excluded from standard clinical laboratory tests, and their high cost further exacerbates the financial burden on patients. Therefore, there is an urgent need to identify a convenient and cost-effective inflammatory-nutritional biomarker that acts as a superior independent predictor of prognosis in patients with CKD stages G3a to G5.

The neutrophil percentage-to-albumin ratio (NPAR) is an emerging biomarker for inflammatory-nutrition, calculated using neutrophil percentage and albumin levels [9]. NPAR provides a comprehensive reflection of inflammatory and nutritional status of individual. Recent studies have demonstrated that the NPAR is a valuable prognostic biomarker for hypertension [10], cardiogenic shock and myocardial infarction [11]. It is also associated with acute kidney injury in patients without chronic kidney disease undergoing percutaneous coronary intervention [12]. No studies have yet elucidated the predictive role of NPAR in all-cause and CVD mortality among patients with CKD stages G3a to G5.

The prognostic effects of NPAR in patients with CKD stages G3a to G5 remain unknown. Therefore, we hypothesize that NPAR levels reflect the inflammatory and nutritional status in patients with CKD G3a to G5 and possess an independent predictive value for all-cause and CVD mortality in this population. This hypothesis aims to offer evidence supporting the timely evaluation and intervention of inflammation and nutritional status in individuals with CKD. To substantiate our hypothesis, we used a large dataset from the National Health and Nutrition Examination Survey (NHANES).

## Materials and methods

### Study populations

The NHANES is a program designed to evaluate the health and nutritional status of both adults and children across the United States. In this study, we utilized data from the NHANES, encompassing survival data procured from the National Centers for Health Statistics (NCHS). This study adhered to the STROBE guidelines for the reporting of observational studies and received approval from the National Center for Health Statistics Research Ethics Review Board. All participants provided written informed consent. Detailed information on the NHANES study design and data is available at https://www.cdc.gov/nchs/nhanes/.

**Figure 1.**
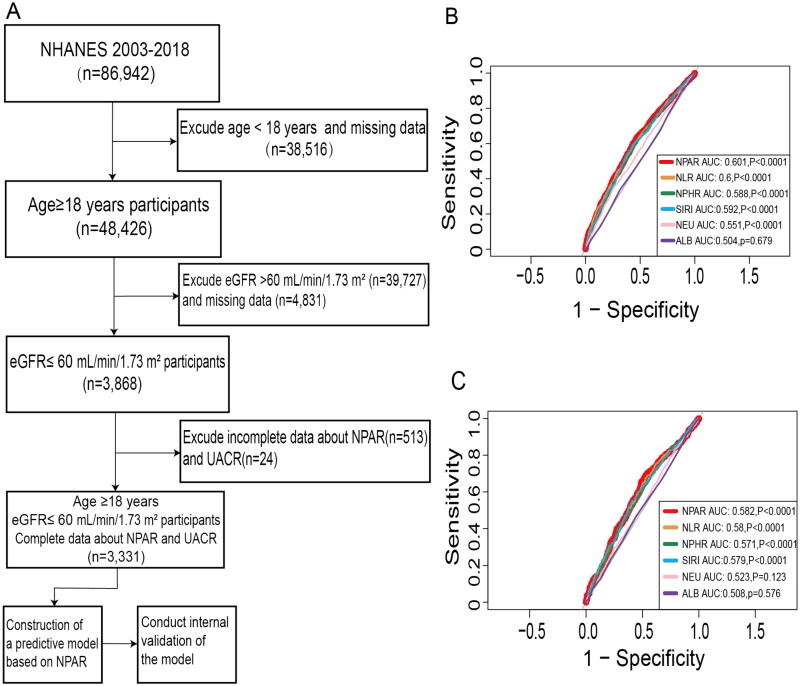
Flow diagram and ROC curves for predictors of all-cause and cardiovascular mortality. A: Flowchart of study inclusion and exclusion. B: ROC curves of inflammatory markers for predicting all-cause mortality in CKD stages G3a to G5. C: ROC curves of inflammatory markers for predicting cardiovascular mortality in CKD stages G3a to G5. Abbreviations: NHANES, National Health and Nutrition Examination Survey; eGFR, estimated glomerular filtration rate; NPAR, neutrophil percentage-to-albumin ratio; UACR, urinary albumin-to-creatinine ratio; NLR, neutrophil-to-lymphocyte ratio; NPHR, neutrophil-to-hemoglobin ratio; SIRI, systemic inflammation response index; NEU, neutrophil; ALB, serum albumin.

We obtained data from the NHANES website covering the period from 2003 to 2018. The study encompassed a total of 86,942 participants. The inclusion criteria were as follows: (i) age ≥18 years; (ii) diagnosis of CKD at stages G3a to G5, defined as an estimated glomerular filtration rate (eGFR) of less than 60 mL/min/1.73 m^2^. The exclusion criteria were: (i) incomplete data regarding neutrophil percentage and albumin levels; (ii) incomplete data on sex, ethnicity, and serum creatinine (SCr) levels; (iii) incomplete data on urinary albumin-to-creatinine ratio (UACR). After excluding participants under the age of 18 (*n* = 38,516), those not in CKD stages G3a-G5 or with missing eGFR data (*n* = 44,558), as well as those with incomplete data on neutrophil percentage and albumin (*n* = 513), and UACR (*n* = 24), a total of 3,331 participants were included in this study (Figure 1(A)).

### Definitions and formulas

NPAR was calculated as the neutrophil percentage divided by serum albumin concentration. UACR was determined as urinary albumin divided by creatinine concentration, and categorized as: normal (<30 mg/g), moderate (30–300 mg/g), severe (300–1000 mg/g), and marked (≥1000 mg/g). eGFR was estimated using the 2009 CKD Epidemiology Collaboration (CKD-EPI) equation, which incorporates SCr, age, race, and sex [13], calculated as: estimated GFR = 175 × (standardized SCr) ^−1.154^ × (age)^−0.203^× 1.212 [if Black] × 0.742 [if female]. GFR is reported in mL/min/1.73 m^2^, and SCr is expressed in mg/dL. eGFR was categorized into G3a (45–60 mL/min/1.73 m^2^), G3b (30–44 mL/min/1.73 m^2^), G4 (15–29 mL/min/1.73 m^2^), and G5 (<15 mL/min/1.73 m^2^) [2^,^]. Dialysis status was ascertained through based on a ‘yes’ response to the question: ‘In the past 12 months, have you received dialysis (either hemodialysis or peritoneal dialysis)?’ in conjunction with an eGFR of less than 60 mL/min/1.73 m^2^. In this study, we also evaluated and analyzed several complex systemic inflammation indices. The neutrophil-to-lymphocyte ratio (NLR), monocyte-to-lymphocyte ratio (MLR) and platelet-to-lymphocyte ratio (PLR) were calculated by dividing the neutrophil count (×10^9^/L), monocyte count (×10^9^/L) and platelet count (×10^9^/L) by lymphocyte count (×10^9^/L), respectively, and expressing these as ratios. The neutrophil-to-hemoglobin ratio (NPHR) was calculated as the neutrophil percentage divided by the hemoglobin concentration. The systemic inflammation response index (SIRI) was computed as the product of the neutrophil and monocyte count, divided by the lymphocyte count. Hypertension status was determined based on a self-reported medical history of high blood pressure, the use of antihypertensive medication, or three separate nonconsecutive records of systolic blood pressure ≥140 mmHg or diastolic blood pressure ≥90 mmHg. Diabetes status was established through a physician’s diagnosis, glycohemoglobin (HbA1c) levels of 6.5% or higher, fasting serum glucose levels of at least 7 mmol/L, two-hour post-load glucose (OGTT) levels of 11.1 mmol/L or greater, or the use of hypoglycemic medications.

### Study variables

For the exploratory analysis, demographic, examination, laboratory, questionnaire, and survival data were systematically collected. Age was categorized into two groups using 65 years as the threshold. Race was classified as Mexican American, other Hispanic, non-Hispanic White, non-Hispanic Black, or other races. The categorizations of UACR and eGFR followed the definitions outlined earlier. We collected demographic data, including age, gender, race, and annual family income (AFI); body measurements such as body mass index (BMI), waist circumference, and arm circumference; and laboratory data covering white blood cell count (WBC), lymphocytes (LYM), monocytes (MON), neutrophils (NEU), platelets (PLT), red blood cell count (RBC), hemoglobin (Hb), aspartate aminotransferase (AST), serum albumin (ALB), alkaline phosphatase (ALP), cholesterol (Chol), potassium (K), sodium (Na), chloride (Cl), creatinine (Cr), blood urea nitrogen (BUN), glucose, iron, uric acid (UA), total calcium (Ca), and phosphorus (P). Questionnaire and survival data were sourced from the NHANES website.

### Ascertainment of mortality

Survival time, vital status, and causes of death were obtained from the National Death Index (NDI), with records provided by the NCHS. Mortality data were updated through December 31, 2019. All-cause mortality included deaths from any causes. CVD mortality was defined as deaths due to major cardiovascular and cerebrovascular diseases, classified according to the International Classification of Diseases, Tenth Revision (ICD-10) codes I00–I09, I11, I13, I20–I51, and I60–I69. Non-cardiovascular (Non-CVD) mortality referred to deaths from causes other than cardiovascular or cerebrovascular diseases. Diabetes mortality was characterized by deaths attributed to diabetes, according to ICD-10 codes E10–E14.

### Statistical analysis

R software version 4.4.1 (R Center for Statistical Computing, Vienna, Austria) was used to perform the statistical analyses. In accordance with the NHANES analytic and reporting guidelines, all analyses incorporated sample weights, clustering, and stratification to ensure nationally representative estimates [14]. Receiver operating characteristic (ROC) curve analyses were conducted to ascertain the predictive ability of NPAR and other composite inflammatory indices for all-cause, CVD, non-CVD, and diabetes-related mortality in participants with CKD stages G3a to G5. The optimal NPAR value was determined using the Youden index. At baseline, NPAR was categorized into two groups based on the optimal cutoff value (14.512) established by the ROC curve, to evaluate differences in the distribution of variables between these groups. Continuous variables were expressed as mean ± standard deviation (SD), non-normally distributed variables as median and interquartile range, and categorical variables as counts (percentage, %). The comparison of continuous variables employed the Student’s t-test, while categorical variables were compared using the χ^2^ test. Standardized mean difference (SMD) was also used to assess differences between variables. Pearson correlation analysis was employed to assess the correlation between NPAR and related variables, with a *p* value of less than 0.05 considered indicative of statistical significance. A heatmap was employed to visually represent these associations.

The log-rank test and Kaplan–Meier (K–M) survival analyses were performed to investigate disparities in event-free survival between the higher and lower NPAR groups. Univariate Cox analysis, least absolute shrinkage and selection operator (LASSO) regression, and multivariate Cox analysis were utilized to identify independent influencing factors and to construct multivariate Cox regression models. Cox proportional hazards models were used to calculate hazard ratios (HR) and 95% confidence intervals (CI) in order to evaluate the associations between different NPAR levels and all-cause and cause-specific mortality in participants with CKD stages G3a to G5. NPAR was divided into two groups based on the optimal value of 14.512, and further categorized into four distinct levels [Q1 (<13.023), Q2 (13.023–14.815), Q3 (14.815–16.656), and Q4 (≥16.656)] using the interquartile range, to enhance the model’s dose sensitivity. Based on the Cox models, restricted cubic spline (RCS) regression models were employed to visualize the models and to detect the non-linear relationship between NPAR and all-cause, CVD or non-CVD mortality. We conducted a rigorous internal validation of the Cox regression model using bootstrap resampling, calibration curves, time-dependent receiver operating characteristic (Time-ROC) analysis, the concordance index (C-index), and a prognostic risk model based on NPAR. This comprehensive evaluation assessed the model’s accuracy, sensitivity, predictive ability, and performance across various time intervals in NPAR’s prognostic ability for mortality. Subgroup analyses were conducted to evaluate the impact of NPAR on all-cause mortality across various subgroups, including those stratified by age (≤ 65 years or >65 years), sex, BMI categories (<18, 18–25, >25), race (Mexican American, other Hispanic, non-Hispanic white, non-Hispanic black, and other races), UACR levels (<30 mg/g, 30–300 mg/g, 300–1000 mg/g, ≥1000 mg/g), eGFR stages (G3a, G3b, G4, G5), hypertension, diabetes status, and dialysis. Statistical significance was defined as *p* < 0.05. Propensity score analysis was used to assess the sensitivity of the model.

## Results

### Baseline characteristics of NPAR in participants with CKD at stages G3a to G5

The study encompassed a total of 3,331 participants. Specifically, there were 1,456 participants categorized in the lower NPAR (<14.512) group within this study, while the remaining 1,875 participants belonged to the higher NPAR (≥14.512) group.

The average age of participants with CKD stages G3a-G5 was 72 years, with 52% being female. At baseline, individuals in lower NPAR group tended to be younger (mean age: 71 years), had a smaller waist circumference (102.62 cm), longer arm circumference (32.74 cm), and lower levels of WBC (7.48 × 10^9^/L), NEU (3.68 × 10^9^/L), ALP (70.63 U/L), UA (6.48 mg/dL), BUN (21.39 μmol/L), Cr (1.47 mg/dL), and K (4.16 mmol/L) compared to those in the higher NPAR group. They also exhibited lower levels of complex systemic inflammation indices, including NLR (1.66), PLR (109.02), MLR (0.29), NPHR (3.9), and SIRI (1.04), than participants with higher NPAR levels. Mexican Americans and non-Hispanic Whites exhibited higher NPAR levels compared to other racial groups. Higher NPAR was associated with lower RBC (4.32 × 10^12^/L), Hb (13.23 g/dL), AST (24.23 U/L), Chol (182.63 mg/dL), ALB (3.94 mg/dL), Ca (9.34 mg/dL), and iron (13.22 mg/dL) levels. Participants with UACR levels above 30 mg/g appeared to have higher NPAR. Notably, NPAR levels were significantly elevated in individuals with CKD stages G3a to G5. Additionally, participants with diabetes or those receiving dialysis also showed elevated NPAR levels (Table 1). The standardized mean difference (SMD) values for sex (0.011), AFI (0.029), BMI (0.095), Arm (0.047), WBC (0.027), PLT (0.057), P (0.026), Na (0.019), Cl (0.016), and the number of individuals with hypertension (0.012) were all below 0.1, indicating minimal differences between the groups. The SMD values for the remaining variables were all above 0.1 in baseline. In addition, the values of UACR (0.344), NEU (0.737), ALP (0.350), ALB (0.828), Ca (0.327), BUN (0.304), NLR (1.412), MLR (0.818), PLR (0.858), NPHR (1.576), and SIRI (1.024) were above 0.3, suggesting significant differences between the NPAR groups (Table S3).

**Table 1.** Characteristics of overall patients and stratified by NPAR.

Characteristic	Overall participants (*n* = 3,331)	NPAR <14.512 (*n* = 1,456)	NPAR ≥14.512 (*n* = 1,875)	*p* value^a^
Age, Mean (SD)	72.45 (10.26)	71.65 (10.06)	73.08 (10.36)	<0.001
Sex, n (%)				0.8
Male	1,593 (48%)	692 (48%)	901 (48%)	
Female	1,738 (52%)	764 (52%)	974 (52%)	
Race, n (%)				<0.001
Mexican American	302 (9.6%)	111 (8.1%)	191 (11%)	
Other Hispanic	165 (5.2%)	86 (6.3%)	79 (4.4%)	
Non-Hispanic White	2,064 (65%)	842 (62%)	1,222 (68%)	
Non-Hispanic Black	623 (20%)	328 (23%)	295 (17%)	
Other race	177 (0.2%)	89 (0.6%)	88(0.5%)	
AFI, Mean (SD)	9.57 (15.19)	9.81 (15.49)	9.37 (14.96)	0.11
BMI, Mean (SD)	29.62 (6.71)	29.27 (6.03)	29.90 (7.20)	0.2
Waist Circ (cm), Mean (SD)	103.60 (14.99)	102.62 (14.11)	104.42 (15.64)	0.007
Arm Circ (cm), Mean (SD)	32.60 (5.14)	32.74 (4.90)	32.50 (5.34)	0.046
UACR (mg/g), n (%)				<0.001
Normal (<30)	2,062 (66%)	1,022 (73%)	1,040 (60%)	
Moderate (30–300)	738 (23%)	296 (21%)	442 (25%)	
Severe (300–1000)	179 (5.7%)	63 (4.5%)	116 (6.7%)	
Marked (≥1000)	164 (5.2%)	25 (1.8%)	139 (8.0%)	
eGFR (mL/min/1.73 m²), n (%)				<0.001
G3a	2,137 (64%)	1,043 (72%)	1,094 (58%)	
G3b	829 (25%)	303 (21%)	526 (28%)	
G4	263 (7.9%)	80 (5.5%)	183 (9.8%)	
G5	102 (3.1%)	30 (2.1%)	72 (3.8%)	
WBC (×10^9^/L), Mean (SD)	7.60 (7.87)	7.48 (11.39)	7.70 (3.07)	<0.001
LYM (×10^9^/L), Mean (SD)	2.17 (6.84)	2.86 (10.29)	1.62 (0.58)	<0.001
MON (×10^9^/L), Mean (SD)	0.62 (0.25)	0.63 (0.28)	0.61 (0.21)	0.6
NEU (×10^9^/L), Mean (SD)	4.53 (2.25)	3.68 (1.44)	5.19 (2.52)	<0.001
RBC (×10^12^/L), Mean (SD)	4.39 (0.56)	4.45 (0.53)	4.34 (0.57)	<0.001
Hb (g/dL), Mean (SD)	13.39 (1.64)	13.59 (1.51)	13.23 (1.71)	<0.001
PLT (×10^9^/L), Mean (SD)	233.47 (73.00)	231.15 (67.86)	235.27 (76.73)	0.3
AST (U/L), Mean (SD)	24.88 (12.57)	25.73 (10.91)	24.23 (13.68)	<0.001
ALP (U/L), Mean (SD)	76.26 (29.53)	70.63 (23.64)	80.62 (32.74)	<0.001
Chol (mg/dL), Mean (SD)	187.76 (45.44)	194.37 (45.35)	182.63 (44.85)	<0.001
ALB (mg/dL), Mean (SD)	4.06 (0.37)	4.22 (0.29)	3.94 (0.37)	<0.001
Ca (mg/dL), Mean (SD)	9.44 (0.46)	9.52 (0.41)	9.37 (0.49)	<0.001
Iron (mg/dL), Mean (SD)	13.85 (5.20)	14.67 (4.89)	13.22 (5.34)	<0.001
P (mg/dL), Mean (SD)	3.81 (0.66)	3.82 (0.60)	3.81 (0.70)	0.055
UA (mg/dL), Mean (SD)	6.57 (1.65)	6.48 (1.53)	6.64 (1.74)	0.009
BUN (umol/L), Mean (SD)	23.71(10.63)	21.93 (9.32)	25.09 (11.36)	<0.001
Cr (mg/dL), Mean (SD)	1.56 (1.16)	1.47 (1.02)	1.63 (1.26)	<0.001
Na (mmol/L), Mean (SD)	139.66 (2.84)	139.69 (2.67)	139.63 (2.96)	0.6
K (mmol/L), Mean (SD)	4.21 (0.46)	4.16 (0.45)	4.24 (0.47)	<0.001
Cl (mmol/L), Mean (SD)	103.15(3.81)	103.18 (3.47)	103.12(4.06)	0.9
NLR, Mean (SD)	2.69 (1.63)	1.66 (0.55)	3.48 (1.74)	<0.001
PLR, Mean (SD)	137.45(65.32)	109.02 (45.55)	159.52 (69.69)	<0.001
MLR, Mean (SD)	0.36 (0.17)	0.29 (0.13)	0.41 (0.18)	<0.001
NPHR, Mean (SD)	4.59 (1.00)	3.90 (0.70)	5.13 (0.86)	<0.001
SIRI, Mean (SD)	1.67 (1.27)	1.04 (0.56)	2.17 (1.44)	<0.001
Hypertension, n (%)	2,419 (73%)	1,053 (72%)	1,366 (73%)	0.7
Diabetes, n (%)	1,010 (30%)	368 (25%)	642 (34%)	<0.001
Dialysis, n (%)	90 (2.7%)	26 (1.8%)	64 (3.4%)	0.004

^a^*p* values were calculated using either Student’s t-test or the chi-square test.

*Abbreviations:* SD, standard deviation; NPAR, Neutrophil-to-Albumin Ratio; AFI, annual family income; BMI, body mass index; Waist Circ, waist circumference; Arm Circ, arm circumference; UACR, urinary albumin-to-creatinine ratio; eGFR, estimated glomerular filtration rate; WBC, white blood cell count; LYM, lymphocyte number; MON, monocyte number; NEU, neutrophil; RBC, red blood cell; Hb, hemoglobin; PLT, platelet; AST, aspartate aminotransferase; ALP, alkaline phosphatase; ALB, serum albumin; Chol, cholesterol; Ca, calcium; P, phosphorus; UA, uric acid; BUN, blood urea nitrogen; Cr, creatinine; Na, sodium; K, potassium; Cl, chloride; NLR, neutrophil-to-lymphocyte ratio; PLR, platelet-to-lymphocyte ratio; MLR, monocyte-to-lymphocyte ratio; NPHR, neutrophil-to-hemoglobin ratio; SIRI, systemic inflammation response index.

**Figure 2.**
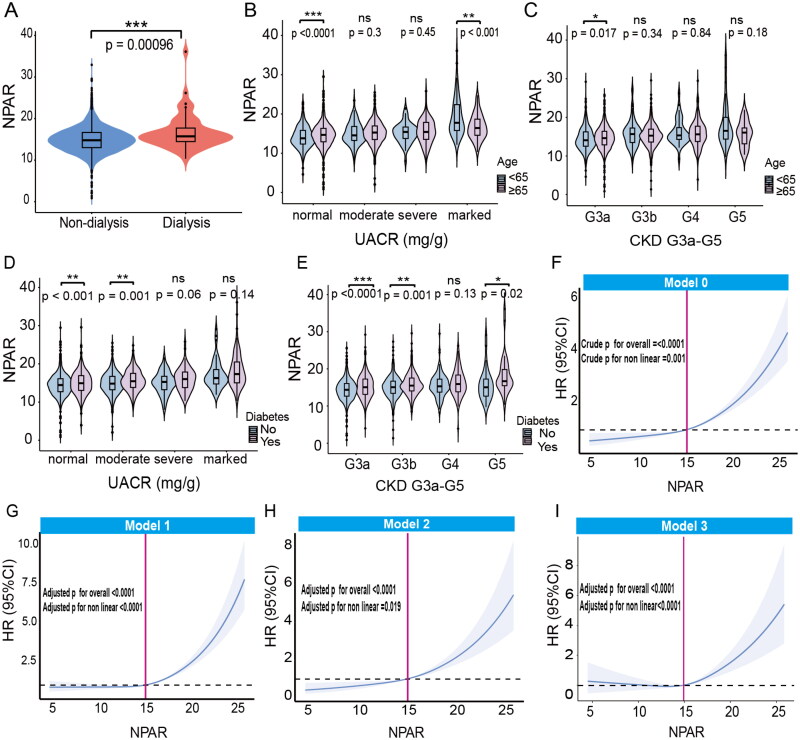
Distribution of NPAR across different subgroups and strata, and RCS for NPAR and all-cause mortality. A: The distribution of NPAR in dialysis and non-dialysis participants. B: The distribution of NPAR across different UACR groups in CKD participants, categorized by age (<65 and ≥65). C: The distribution of NPAR across different eGFR stages within the CKD participants, categorized by age (<65 and ≥65). D: The distribution of NPAR across different UACR groups in diabetic and non-diabetic CKD participants. E: The distribution of NPAR across different eGFR stages in diabetic and non-diabetic CKD participants. F-I: RCS for NPAR and all-cause mortality in CKD stages G3a to G5 adjusted by different models. Abbreviations: NPAR, neutrophil percentage-to-albumin ratio; UACR, urinary albumin-to-creatinine ratio; eGFR, estimated glomerular filtration rate; HR, hazard ratio; CI, confidence interval.

Based on the baseline analysis, we further examined participants undergoing dialysis and those not receiving dialysis. We discovered that individuals on dialysis exhibited a significantly higher NPAR levels compared to those who not on dialysis (*p* < 0.001) (Figure 2(A)). Upon analyzing the stratification of the relationship between NPAR and UACR, our findings revealed that participants younger than 65 exhibited lower NPAR levels in the normal UACR group (<30 mg/g) but higher NPAR levels in the marked UACR group (≥1000 mg) (Figure 2(B)). In individuals with diabetes, NPAR levels were elevated in both the normal UACR (<30 mg/g) and the moderate UACR (30–300 mg/g) groups (Figure 2(D)). The analysis of the correlation between NPAR and different CKD stages revealed that individuals aged 65 years or older presented with an higher NPAR levels at CKD stage G3a (Figure 2(C)). Furthermore, individuals with diabetes demonstrated increased NPAR levels at CKD stages G3a, G3b, and G5 (Figure 2(E)).

### The correlation analysis between NPAR and clinical characteristics of CKD at stages G3a to G5

Pearson correlation analysis was performed to examine the relationship between NPAR and various clinically relevant parameters among participants with CKD stages G3a-G5, and a heatmap was generated to visually represent the results. NPAR exhibited a positive correlation with BMI (*r* = 0.08, *p* < 0.0001), NEU (*r* = 0.41, *p* < 0.0001), PLT (*r* = 0.09, *p* < 0.0001), NLR (*r* = 0.73, *p* < 0.0001), PLR (*r* = 0.51, *p* < 0.0001), MLR (*r* = 0.43, *p* < 0.0001), NPHR (*r* = 0.79, *p* < 0.0001), SIRI (*r* = 0.56, *p* < 0.0001), ALP (*r* = 0.17, *p* < 0.0001), BUN (*r* = 0.19, *p* < 0.0001), Cr (*r* = 0.13, *p* < 0.0001), UA (*r* = 0.08, *p* < 0.0001), and K (*r* = 0.08, *p* < 0.0001). Conversely, it was negatively correlated with MON (r=-0.12, *p* < 0.0001), LYM (r=-0.2, *p* < 0.0001), RBC (r=-0.12, *p* < 0.0001), Hb (r=-0.14, *p* < 0.0001), ALB(r=-0.54, *p* < 0.0001), AST (r=-0.07, *p* < 0.0001), Ca (r=-0.26, *p* < 0.0001), Chol (r=-0.12, *p* < 0.0001), and iron (r=-0.18, *p* < 0.0001) (Figure S1(C)).

**Figure 3.**
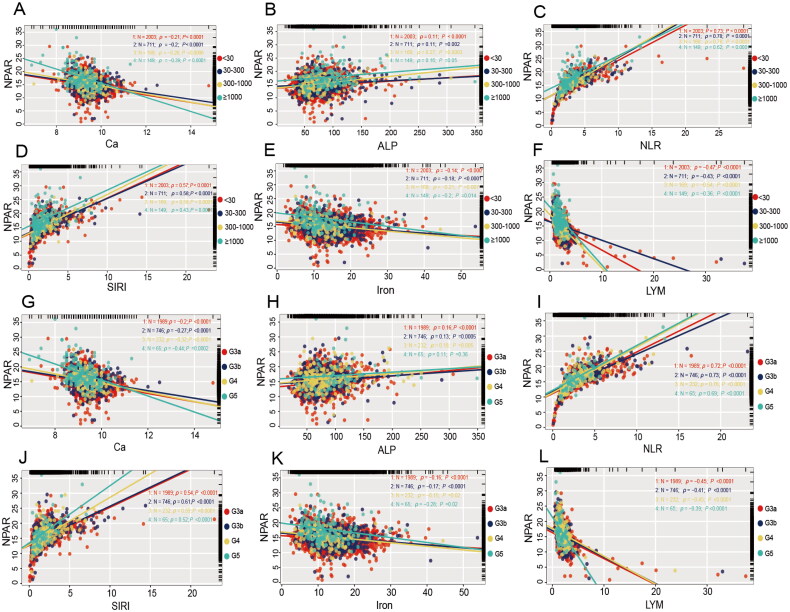
Pearson stratified correlation analysis of NPAR and related-factors. Subgroup by UACR: A: calcium (Ca); B: alkaline phosphatase (ALP); C: neutrophil-to-lymphocyte ratio (NLR); D: systemic inflammation response index (SIRI); E: iron; F: lymphocyte (LYM). Subgroup by eGFR: G: calcium (Ca); H: alkaline phosphatase (ALP); I: neutrophil-to-lymphocyte ratio (NLR); J: systemic inflammation response index (SIRI); K: iron; L: lymphocyte (LYM). Abbreviations: NPAR, neutrophil percentage-to-albumin ratio; UACR, urinary albumin-to-creatinine ratio; eGFR, estimated glomerular filtration rate.

We conducted a stratified Pearson correlation analysis across various UACR levels (< 30 mg/g, 30–300 mg/g, 300–1000 mg/g, ≥1000 mg/g) and CKD stages (G3a, G3b, G4, G5). The results indicated that ALP, NLR, and SIRI were positively correlated within these UACR and CKD categories. In contrast, the stratified analysis relationship between NPAR and Ca, iron, and LYM, revealed a significant negative correlation across the different UACR levels and CKD stages (Figure 3(A–L)).

### The superior prognostic ability of NPAR in predicting mortality

Multiple ROC curves were generated to evaluate the predictive capabilities of NPAR, NLR, NPHR, SIRI, NEU, and ALB for mortality. For all-cause mortality, the area under the curve (AUC) for NPAR was 0.601 (Figure 1(B)), which was higher than that of the other markers. However, no statistically significant differences were observed when comparing NPAR with NLR, NPHR, and SIRS using the DeLong test. Nonetheless, NPAR outperformed NEU [0.601 (95% CI 0.581–0.622) vs. 0.55 (95% CI 0.53–0.571), *p* < 0.0001] and ALB [0.504 (95% CI 0.483–0.525), *p* = 0.679] when assessed individually. This suggests that NPAR offers superior predictive value for mortality compared to NEU or ALB alone. Notably, NPAR exhibited substantial predictive potential for all-cause mortality, with an optimal cutoff value of 14.512, a sensitivity of 60.1%, and a specificity of 43.6%, resulting in a maximum Youden’s index of 0.165 (Table S1).

Furthermore, NPAR demonstrated remarkable and robust prognostic abilities in predicting both CVD mortality (Figure 1(C)) and non-CVD mortality (Figure S1(A)). Notably, for predicting diabetes mortality, the AUC for NPAR stood at 0.631 (Figure S1(B)), which showed statistically significant differences from NLR, SIRI, and NEU as assessed by the DeLong test. This underscores the unique advantage of NPAR in predicting diabetes mortality among individuals with CKD stages G3a to G5 (Table S1).

**Figure 4.**
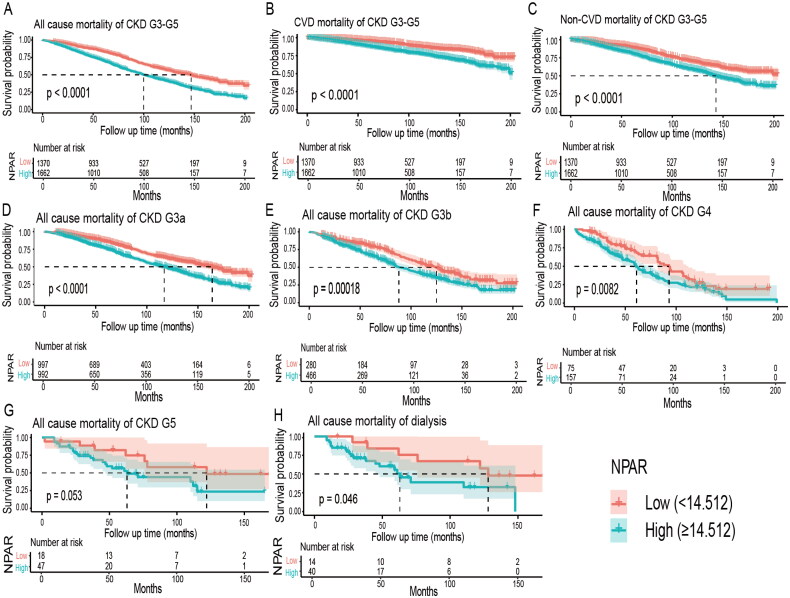
The Kaplan–Meier survival curves of overall, CVD, non-CVD, and stage-specific survival. A: Overall patients; B: CVD mortality in CKD G3a to G5; C: non-CVD mortality in CKD G3a to G5; D: CKD G3a; E: CKD G3b; F: CKD G4; G: CKD G5; H: patients on dialysis. Abbreviations: CKD, chronic kidney disease; CVD, cardiovascular disease; non-CVD, non-cardiovascular disease; NPAR, neutrophil percentage-to-albumin ratio.

Over an average follow-up period of 79.72 months, a total of 1,352 deaths from all causes were documented, resulting in an all-cause mortality rate of 1,555 per 100,000 person-years. Additionally, 423 deaths due to cardiovascular causes were recorded, yielding a cardiovascular mortality rate of 586 per 100,000 person-years. Furthermore, 167 deaths attributed to diabetes were recorded, resulting in a diabetes mortality rate of 192 per 100,000 person-years. Kaplan-Meier survival curves were used to analyze the relationship between different levels of NPAR (Low group <14.512, High group ≥ 14.512) and distinct survival rates among participants with CKD stages G3a to G5. The analyses revealed a significant disparity in the occurrence of all-cause, CVD and non-CVD mortality between the two NPAR groups (log-rank *p* < 0.0001, Figure 4(A–C)). Furthermore, we assessed survival rates across different stages of CKD and NPAR groups. Except for those in the G5 category, individuals with CKD at stages G3a, G3b, G4, as well as those undergoing dialysis, all exhibited statistically significant disparities in their all-cause survival probabilities across varying NPAR levels (log-rank *p* < 0.05, Figure 4(D–H)). Higher NPAR levels were associated with lower CVD survival rates across different CKD stages and dialysis status, with the exception of stage G4 (Figure S2(C–G)). Additionally, participants with lower NPAR levels exhibited higher survival rates related to diabetes (log-rank *p* < 0.0001, Figure S2(B)), while no significant differences were observed in infection-related mortality (*p* = 0.49, Figure S2(A)).

### Higher NPAR stood out as an independent risk factor in CKD at stages G3a to G5

**Figure 5.**
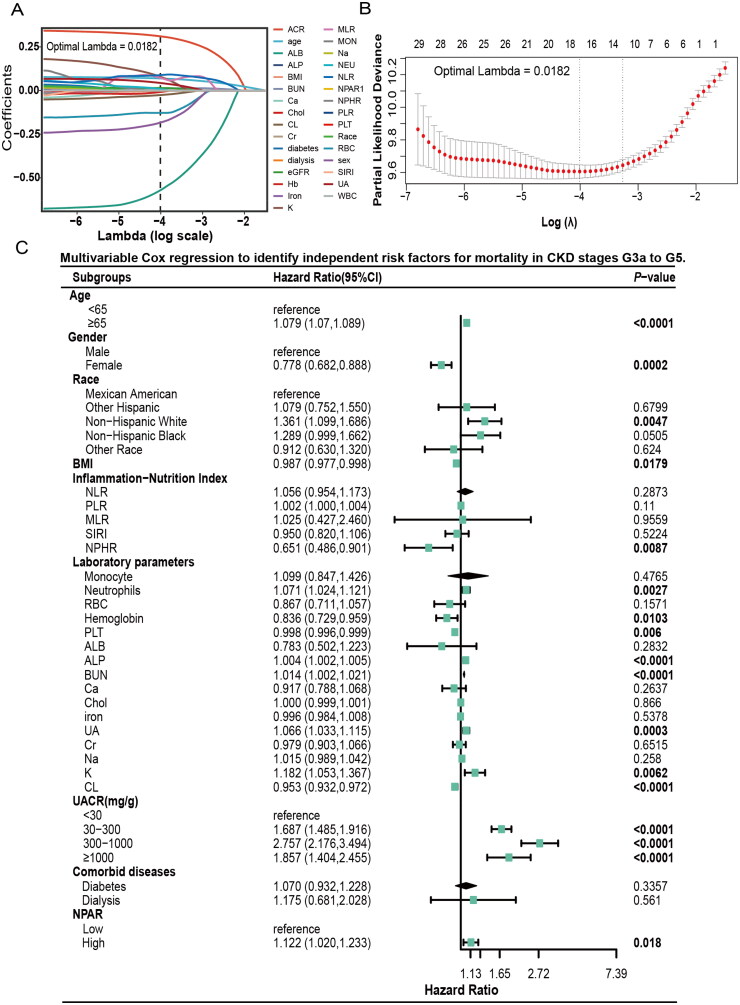
LASSO regression and multivariable Cox regression. A-B: LASSO regression analysis of prognosis-associated factors in CKD stages G3a to G5. C: Multivariable Cox regression to identify independent risk factors for mortality in CKD stages G3a to G5. Abbreviations: CI, Confidence Interval; BMI, body mass index; NLR, neutrophil-to-lymphocyte ratio; PLR, platelet-to-lymphocyte ratio; MLR, monocyte-to-lymphocyte ratio; SIRI, systemic inflammation response index; NPHR, neutrophil-to-hemoglobin ratio; RBC, red blood cell; PLT, platelet; ALB, serum albumin; BUN, blood urea nitrogen; Ca, calcium; Chol, cholesterol; UA, uric acid; Cr, creatinine; Na, sodium; K, potassium; Cl, chloride; UACR, urinary albumin-to-creatinine ratio; NPAR, neutrophil percentage-to-albumin ratio.

Univariate Cox regression analysis was conducted to identify variables associated with all-cause mortality (Figure S3). Variables identified through univariate Cox regression analysis were included in the LASSO regression for further selection. As illustrated in Figure 5(A,B), the optimal lambda min value for the LASSO regression was 0.0182. The 17 variables identified by LASSO regression included age, sex, BMI, NEU, RBC, Hb, PLT, NLR, MLR, ALB, ALP, BUN, UA, eGFR, K, Cl, and UACR. These variables, along with other clinically relevant factors related to overall mortality in patients with CKD stages G3a-G5, were subsequently subjected to multivariate Cox analysis to further identify predictors. The multivariate Cox regression analysis results indicated that higher NPAR (HR 1.122; 95% CI 1.020–1.233; *p* = 0.018), age over 65 years (HR 1.079; 95% CI 1.07–1.089; *p <* 0.0001), being non-Hispanic White (HR 1.377; 95% CI 1.069–1.773; *p* = 0.013) or non-Hispanic Black (HR 1.289; 95% CI 0.999–1.662; *p* = 0.05), elevated NEU (HR 1.071; 95% CI 1.024–1.121; *p* = 0.0027), ALP (HR 1.004; 95% CI 1.002–1.005; *p* < 0.0001), BUN (HR 1.014; 95% CI 1.002–1.021; *p* < 0.0001), UA (HR 1.066; 95% CI 1.033–1.115; *p* = 0.0003), K (HR 1.182; 95% CI 1.053–1.367; *p* = 0.0062), and UACR levels classified as moderate (HR 1.687; 95% CI 1.485–1.916; *p* < 0.0001), severe (HR 2.757; 95% CI 2.176–3.494; *p* < 0.0001), or marked (HR 1.857; 95% CI 1.404–2.455; *p* < 0.0001) were independent risk factors for increased all-cause mortality. It is noteworthy that within the complex inflammation indices, NLR, PLR, MLR, and SIRI did not emerge as independent predictors of mortality risk. Nevertheless, higher NPAR (HR 1.122; 95% CI 1.020–1.233; *p* = 0.018) was identified as an independent risk factor for mortality participants with CKD at stages G3a to G5. Factors associated with a protective effect included being female (HR 0.778; 95% CI 0.682–0.888; *p* = 0.0002), high NPHR level (HR 0.651; 95% CI 0.486–0.901; *p* = 0.00187), Hb (HR 0.836; 95% CI 0.729–0.959; *p* = 0.0103), PLT (HR 0.998; 95% CI 0.996–0.999; *p* = 0.006), and Cl (HR 0.953; 95% CI 0.932–0.972; *p <* 0.0001) (Figure 5(C)).

Employing a combination of univariate Cox analysis, LASSO regression, and multivariate Cox analysis, 28 variables were selected to construct Cox regression models. Different adjustment models were developed to minimize clinical bias: Model 0 was unadjusted for any covariates; Model 1 accounted for age, sex, and race; Model 2 included adjustments for age, sex, race, NEU, Hb, PLT, ALP, BUN, Ca, UA, K, Cl, NPHR, and UACR; and Model 3 was adjusted for age, sex, race, BMI, UACR, MON, NEU, RBC, Hb, PLT, NLR, PLR, MLR, NPHR, SIRI, ALP, BUN, Ca, Chol, iron, UA, Cr, Na, K, Cl, diabetes, and dialysis. Table 2 presents the four Cox regression models employed to assess the association between NPAR and all-cause mortality. The variables adjusted in constructing these models have been mentioned above. NPAR was analyzed both as a continuous variable and by categorizing it into high and low groups. Additionally, NPAR was stratified into quartiles to further investigate its effects, and the models was tested across different CKD stages to evaluate sensitivity. Continuous NPAR was associated with a worse prognosis among participants with CKD stages G3a-G5 [Model 0: *p* < 0.001, HR = 1.119, 95%CI: (1.099–1.139); Model 1: *p* < 0.001, HR = 1.132, 95% CI: (1.11–1.154); Model 2: *p* < 0.001, HR = 1.085, 95% CI: (1.052–1.118); Model 3: *p* < 0.001, HR = 1.179, 95% CI: (1.134–1.227)]. When NPAR ≥ 14.512, the risk of all-cause mortality increased among participants with CKD G3a-G5 [Model0: *p* < 0.001, HR = 1.719, 95%CI: (1.538–1.921); Model 1: *p* < 0.001, HR = 1.58, 95%CI:(1.412–1.768); Model 2: *p* = 0.009, HR = 1.23,95% CI:(1.053–1.437); Model 3: *p* = 0.01, HR = 1.23, 95% CI: (1.05–1.44)]. When NPAR was categorized into quartiles (Q1: <13.023, Q2:13.023–14.815, Q3: 14.815–16.656, Q4: ≥16.656), participants in Q4 had a significantly worse prognosis compared with the Q1 group (Model 3: *p* = 0.005, HR = 1.627, 95% CI: 1.233–2.147). In different stages of CKD, Cox regression models demonstrated that NPAR was remained an independent prognostic indicator for all-cause mortality in CKD stage G3a and among those undergoing dialysis. Compared with participants with lower NPAR, higher NPAR increased the risk of death in CKD G3a [Model 0: *p* < 0.001, HR =1.671, 95% CI: (1.451–1.925); Model 1: *p* < 0.001, HR = 1.48, 95% CI: (1.280–1.701); Model 2: *p* = 0.029, HR = 1.252, 95% CI: (1.021–1.534); Model 3: *p* = 0.134, HR = 1.17, 95% CI: (0.953–1.437)] and dialysis status [Model 0: *p* = 0.052, HR = 2.584, 95% CI: (0.993–6.723); Model 1: *p* = 0.013, HR = 3.457, 95% CI: (1.294–9.235); Model 2: *p* = 0.006, HR = 27.12, 95% CI: (2.582–284.946); Model 3:*p* < 0.001, HR =172.100, 95% CI: (57.220–517.6)].

**Table 2.** Cox models of NPAR for all-cause mortality in CKD stage G3a-5D participants.

Model 0			Model 1		Model 2		Model 3	
Variables	Crude HR (95% CI)	Crude P	Adjusted HR (95% CI)	Adjusted *P*	Adjusted HR (95% CI)	Adjusted *P*	Adjusted HR (95% CI)	Adjusted *P*
Overall patients								
As continuous (per SD)	1.119 (1.099–1.139)	<0.001	1.132 (1.11–1.154)	<0.001	1.085 (1.052–1.118)	<0.001	1.179 (1.134–1.227)	<0.001
By NPAR cutoff								
NPAR < 14.512	1		1		1		1	
NPAR ≥ 14.512	1.719 (1.538–1.921)	<0.001	1.58 (1.412–1.768)	<0.001	1.23 (1.053–1.437)	0.009	1.23(1.05–1.44)	0.01
By NPAR cutoff								
NPAR ≥ 14.512	1		1		1		1	
NAPR < 14.512	0.583 (0.521–0.651)	<0.001	0.638 (0.57–0.713)	<0.001	0.813 (0.696–0.95)	0.009	0.813 (0.693–0.953)	0.01
By Interquartile								
Q1 (<13.023)	1		1		1		1	
Q2 (13.023–14.815)	1.144 (0.968–1.351)	0.114	0.105 (0.934–1.306)	0.246	1,011 (0.831–1.123)	0.917	1.6 (0.87–1.306)	0.56
Q3 (14.815–16.656)	1.513 (1.2923–1.772)	<0.001	1.325 (1.129–1.554)	<0.001	1.184 (0.95–1.475)	0.133	1.233 (0.979–1.555)	0.075
Q4 (≥16.656)	2.254 (1.933–2.628)	<0.001	2.165 (1.856–2.531)	<0.001	1.559(1.225–2.09)	<0.001	1.627 (1.233–2.147)	0.005
P for trends		<0.001		<0.001		<0.001		<0.001
By eGFR								
CKD G3a								
NPAR < 14.512	1		1		1		1	
NPAR ≥ 14.512	1.671 (1.451–1.925)	<0.001	1.48 (1.28–1.701)	<0.001	1.252 (1.021–1.534)	0.03	1.17 (0.953–1.437)	0.134
CKD G3b								
NAPR < 14.512	1		1		1		1	
NAPR ≥ 14.512	1.513 (1.216–1.882)	<0.001	1.423 (1.143–1.772)	0.002	1.025 (0.751–1.398)	0.878	1.139 (0.822–1.578)	0.435
CKD G4								
NPAR < 14.512	1		1		1		1	
NPAR ≥ 14.512	1.615 (1.127–2.314)	0.009	1.736 (1.2–2.51)	0.003	1.34 (0.788–2.279)	0.28	1.47 (0.83–2.604)	0.187
CKD G5								
NPAR < 14.512	1		1		1		1	
NPAR ≥ 14.512	2.326 (0.9688–5.582)	0.059	4.619 (1.711–12.471)	0.002	2.046 (0.415–10.09)	0.379	11.02 (1.131–107.4)	0.039
Dialysis								
NPAR < 14.512	1		1		1		1	
NPAR ≥ 14.512	2.584 (0.993–6.723)	0.052	3.457 (1.294–9.235)	0.013	27.12(2.582–284.946)	0.006	172.1 (57.22–517.6)	<0.001

*Abbreviations:* HR, hazard ratios; CIs, confidence intervals; NPAR, neutrophil percentage-to-albumin ratio; eGFR, estimated glomerular filtration rate; CKD, chronic kidney disease.

Model 0: unadjusted. Model 1, adjusted for age, sex, and race. Model 2, adjusted for age, sex, race, NEU, Hb, PLT, ALP, BUN, Ca, UA, K, Cl, NPHR, and UACR. Model 3, adjusted for age, sex, race, BMI, UACR, MON, NEU, RBC, Hb, PLT, NLR, PLR, MLR, NPHR, SIRI, ALP, BUN, Ca, Chol, iron, UA, Cr, Na, K, Cl, diabetes, and dialysis.

Additionally, we investigated the prognostic value of NPAR in predicting CVD mortality and non-CVD mortality. The same variables as those used for all-cause mortality were adjusted in the four models constructed for CVD and non-CVD mortality. Continuous NPAR was associated with a worse prognosis for CVD mortality [Model 0: *p* < 0.0001, HR =1.135, 95% CI: (1.100–1.170); Model 1: *p* < 0.0001, HR = 1.153, 95% CI: (1.115–1.193); Model 2: *p* < 0.0001, HR = 1.202, 95% CI: (1.123–1.287); Model 3: *p* < 0.0001, HR = 1.181, 95% CI: (1.100–1.268)]. When NPAR was used as a categorical variable, participants with high NPAR had an increased risk of CVD mortality in CKD stages G3a-G5 compared with those patients with low NPAR [Model 0: *p* < 0.0001, HR =2.105, 95% CI: (1.714–2.585); Model 1: *p* < 0.0001, HR =1.949, 95% CI: (1.583–2.398); Model 2: *p* = 0.001, HR = 1.593, 95% CI: (1.198–2.118); Model 3: *p* = 0.005, HR = 1.513, 95% CI: (1.131–2.024)]. Continuous NPAR was also associated with a worse prognosis of non-CVD mortality [Model 0: *p* < 0.0001, HR =1.120, 95% CI: (1.095–1.145); Model 1: *p* < 0.0001, HR =1.131, 95% CI: (1.104–1.159); Model 2: *p* < 0.0001, HR = 1.178, 95% CI:(1.122–1.238); Model 3: *p* < 0.0001, HR = 1.180, 95% CI: (1.123–1.243)]. No significant association was found when NPAR was utilized as a categorical variable to predict non-CVD mortality (Table S2).

### Detection of non-linear trends of NPAR with all-cause, CVD and non-CVD mortality among participants with CKD G3a to G5

RCS was employed to identify and illustrate the non-linear correlation between the NPAR and mortality rates among participants with CKD stages G3a to G5. After adjusting for age, sex, race, BMI, UACR, MON, NEU, RBC, Hb, PLT, NLR, PLR, MLR, SIRI, ALP, BUN, Ca, Chol, iron, UA, Cr, Na, K, Cl, diabetes, and dialysis, we observed a significant J-shaped association between NPAR and all-cause mortality (Figure 2(F–1)), according to the Cox regression models (all *p* values for nonlinear <0.05). The value of NPAR associated with the lowest risk of all-cause mortality, as determined by multivariate-adjusted RCS, was 14.90. Similarly, a J-shaped relationship was observed between NPAR and both cardiovascular and non-cardiovascular mortality (*p* values for non-linearity < 0.05), even after adjusting for multiple covariates. The lowest risks for CVD and non-CVD mortality were associated with NPAR values of 14.80 and 14.78, respectively (Fig. S4(A–H)).

### The internal validation confirmed the superiority of the NPAR predictive ability for all-cause and CVD mortality in CKD G3a to G5

**Figure 6.**
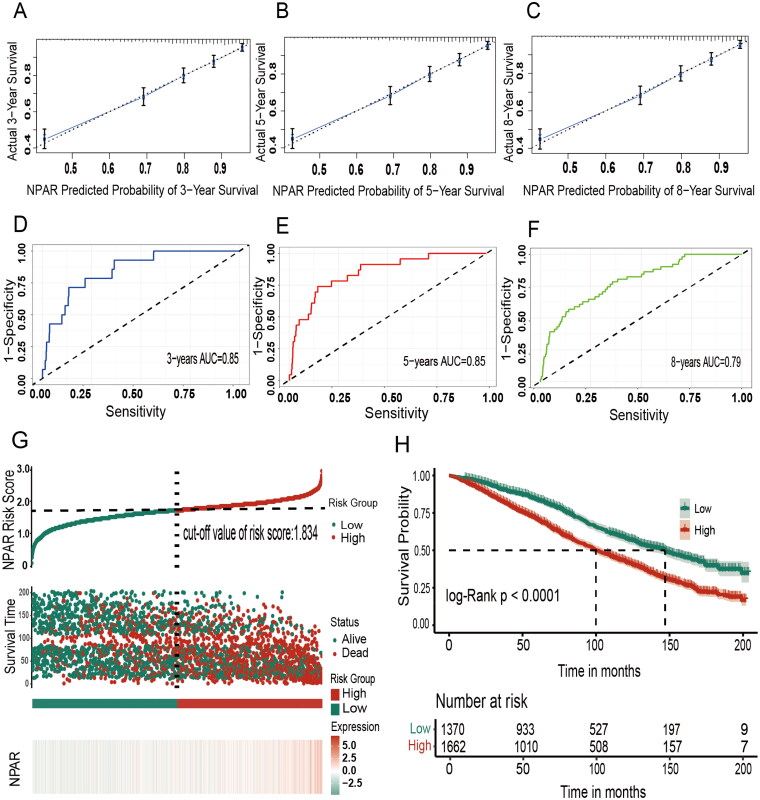
The 3-, 5-, and 8-year calibration curves, time-dependent ROC curves, and the risk assessment model. A–C: 3-, 5-, and 8-year calibration curves of NPAR in CKD stages G3a to G5; D–F: 3-, 5-, and 8-year time-dependent ROC curves of NPAR in CKD stages G3a to G5; G–H: Prognostic risk score model for CKD stages G3a to G5 based on NPAR. Abbreviations: NPAR, neutrophil percentage-to-albumin ratio; AUC, area under the curve.

We divided the data into training and validation sets in a 0.7:0.3 ratio for internal validation of the model. The concordance index (C-index) was utilized to assess the predictive ability of the models. After adjusting for all variables, the model exhibited a C-index of 0.743 (*p* < 0.0001, 95% CI, 0.727–0.759) in the training set and 0.734 (*p* < 0.0001, 95% CI, 0.707–0.761) in the validation set. An increase in NPAR was positively associated with a higher risk of all-cause mortality (*p* < 0.0001, HR = 2.148, 95% CI: 1.956–2.359), and the model demonstrated good predictive performance (C-index = 0.734). The model also showed favorable discrimination for CVD mortality (training set: C-index = 0.755, 95% CI, 0.725–0.788; validation set: C-index = 0.739, 95% CI, 0.687–0.792, all *p* < 0.0001) and non-CVD mortality (training set: C-index = 0.735, 95% CI, 0.713–0.757; validation set: C-index = 0.753, 95% CI, 0.723–0.783, all *p* < 0.0001). These results illustrate that higher NPAR levels were associated with an increased risk of CVD mortality (*p* < 0.0001, HR = 2.337, 95% CI: 1.932–2.828) or non-CVD mortality (*p* < 0.0001, HR = 2.010, 95% CI: 1.823–2.215), and the models exhibited high predictive accuracy (Table 3). The 3-, 5-, and 8-year calibration curve results showed that NPAR had good predictive accuracy for all-cause (Figures 6(A–C) and S5(A–C)) and CVD (Figure S5(G–I)) mortality. Upon evaluating the predictive efficacy of NPAR for all-cause and cardiovascular mortality among CKD G3a-G5 patients through time-dependent ROC analysis, it was determined that NPAR demonstrated superior predictive capacity for both all-cause (Figures 6(D–F) and S^6^(D–F)) and cardiovascular mortality (Figure S5(J–L)) over 3-, 5-, and 8-year spans. NPAR demonstrated high predictive accuracy for overall survival, with AUC values of 0.85 at 3 years, 0.85 at 5 years, and 0.79 at 8 years. Likewise, its predictive accuracy for cardiovascular survival was notable, with AUC values of 0.77, 0.79, and 0.77 at the same intervals.

**Table 3.** Index of concordance for NPAR and all-cause, CVD or non-CVD mortality.

	C-index	C-index change (95%CI)	*p* value
All-cause			
training	0.743	0.008 (0.727–0.759)	<0.0001
validation	0.734	0.014 (0.707–0.761)	<0.0001
	HR	95%CI	*p* value
Predict model	2.148	1.956–2.359	<0.0001
	C-index	C-index change (95%CI)	*p* value
CVD			
training	0.755	0.01 (0.725–0.788)	<0.0001
validation	0.739	0.027 (0.687–0.792)	<0.0001
	HR	95%CI	*p* value
Predict model	2.337	1.932–2.828	<0.0001
	C-index	C-index change (95%CI)	*p* value
Non CVD			
training	0.735	0.01 1(0.713–0.757)	<0.0001
validation	0.753	0.015 (0.723–0.783)	<0.0001
	HR	95%CI	*p* value
Predict model	2.01	1.823–2.215	<0.0001

*Abbreviations*: C-index, the concordance index; CI, confidence interval; HR, hazard ratio; CVD, cardiovascular; NPAR, neutrophil percentage-to-albumin ratio.

We developed a prognostic risk model based on NPAR. The risk score was calculated using the NPAR value and the β regression coefficient, which was derived from the multivariate Cox regression model. The formula is: NPAR risk score = NPAR value × 0.139. Higher NPAR values corresponded to higher risk scores, indicating an elevated prognostic risk. The risk score cutoff value was determined to be 1.834 based on NPAR levels. Additionally, a prognostic heatmap and survival curve were generated according to the risk score. The results demonstrated that participants with higher risk scores had worse survival outcomes compared to those with lower risk scores (Figure 6(G–H)).

### Subgroup analyses of association between NPAR and all-cause mortality among participants with CKD G3a to G5

**Figure 7.**
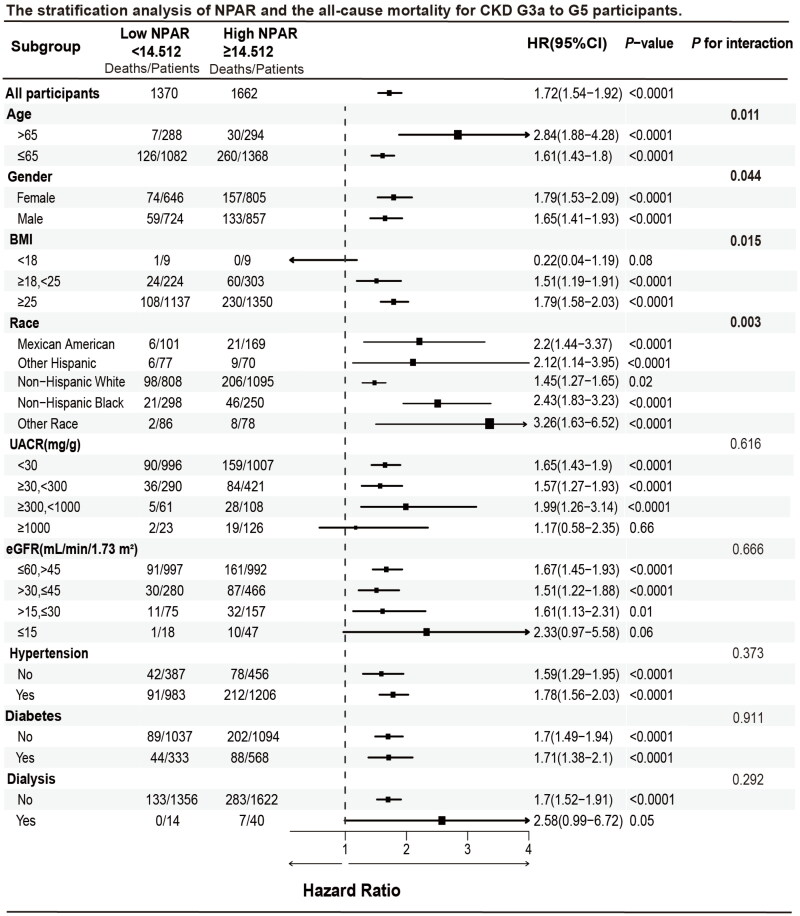
Stratification analysis of NPAR and the all-cause mortality in CKD stages G3a to G5 participants. *Abbreviations:* NPAR, neutrophil percentage-to-albumin ratio; CI, Confidence Interval; BMI, body mass index; UACR, urinary albumin-to-creatinine ratio; eGFR, estimated glomerular filtration rate.

To further investigate the association between NPAR and all-cause mortality in diverse populations, we stratified CKD stages G3a to G5 participants by age, sex, race, UACR, eGFR, hypertension, diabetes, and dialysis status. NPAR was categorized into low (<14.512) and high (≥14.512) groups. Overall, high NPAR was associated with a significantly increased risk of mortality compared to low NPAR (HR = 1.72, 95% CI: 1.54–1.92, *p* < 0.0001). A significantly higher risk of all-cause mortality was observed in participants aged > 65 years (HR = 2.84, 95% CI: 1.88–4.28, *p* < 0.0001) in comparison to those aged ≤ 65 years (HR =1.61, 95% CI:1.43–1.88, *p* < 0.0001), with a significant interaction between age groups (*p* for interaction = 0.011). Additionally, female patients with high NPAR (HR = 1.79, 95% CI: 1.53–2.09, *p* < 0.0001) had a higher mortality risk compared to males (HR = 1.65, 95% CI: 1.41–1.93, *p* < 0.0001), with a significant interaction by sex (*p* for interaction = 0.044). Participants with a BMI ranging from 18 to 25 (HR = 1.51, 95% CI: 1.19–1.91, *p* < 0.0001) and those with a BMI ≥25 (HR = 1.79, 95% CI: 1.58–2.03, *p* < 0.0001) experienced a higher risk of death, with an interaction *p* value of 0.015. Individuals of other races (HR = 3.26, 95% CI: 1.63–6.52, *p* < 0.0001) had a significantly higher risk of death compared to Mexican Americans (HR = 2.2, 95% CI: 1.44–3.37, *p* < 0.0001), other Hispanics (HR = 2.12, 95% CI: 1.14–3.95, *p* < 0.0001), non-Hispanic Whites (HR = 1.45, 95% CI: 1.27–1.65, *p* = 0.02), and non-Hispanic Blacks (HR = 2.43, 95% CI: 1.83–3.23, *p* < 0.0001), with an interaction *p* value of 0.003. Similarly, participants with higher NPAR exhibited an increased risk of death in the UACR <30 group (HR = 1.65, 95% CI: 1.43–1.90, *p* < 0.0001), UACR 30–300 group (HR = 1.57, 95% CI: 1.27–1.93, *p* < 0.0001), and UACR 300–1000 group (HR = 1.99, 95% CI: 1.26–3.14, *p* < 0.0001). The risk was also elevated in the eGFR 45–60 group (HR = 1.67, 95% CI: 1.45–1.93, *p* < 0.0001), eGFR 30–45 group (HR = 1.51, 95% CI: 1.22–1.88, *p* < 0.0001), and eGFR 15–30 group (HR = 1.61, 95% CI: 1.13–2.31, *p* = 0.01). Patients with hypertension (HR = 1.78, 95% CI: 1.56–2.03, *p* < 0.0001) or those undergoing dialysis (HR = 2.58, 95% CI: 0.99–6.72, *p* = 0.05) also exhibited an increased risk of death, although the interaction *p* value was not significant (Figure 7).

### Sensitivity analyses

First, after 1:1 propensity score matching of baseline characteristics, 926 cases in the low NPAR group were matched to 926 cases in the high NPAR group. The SMDs for all variables were less than 0.1, demonstrating good intergroup balance (Table S4). Furthermore, the robustness of the results was confirmed in the post-matching Cox regression models (Table S5). After adjusting for eGFR in both the primary models (Table S6) and the post-matching models (Table S5), these associations remained largely unchanged. Additionally, Cox models were also constructed by stratifying participants into quartiles based on NPAR levels, and the associations were largely consistent (Table 2). Sensitivity analysis confirmed that NPAR remained an independent prognostic indicator for CKD stages G3a to G5.

## Discussion

No studies have yet elucidated the predictive role of NPAR in all-cause and CVD mortality among patients with CKD stages G3a to G5. To our knowledge, this is the first study to investigate the relationship between NPAR and both all-cause and CVD mortality in CKD stages G3a to G5. Utilizing data from the NHANES database in the United States, we discovered that NPAR has significant predictive power for both all-cause and cardiovascular mortality in these patients. Higher NPAR levels was associated with an increased risk of all-cause and cardiovascular mortality. After adjusting for potential confounders, we identified a significant nonlinear J-shaped association between NPAR and both all-cause and CVD mortality. Internal validation confirmed the consistency and accuracy of the mortality risk prediction model based on NPAR for patients with CKD stages G3a to G5. Additionally, we found that NPAR has a significant advantage in predicting diabetes-related mortality risk. NPAR represents a novel and reliable predictive biomarker for CKD stages G3a to G5, capable of accurately assessing the inflammation-nutrition status and providing an opportunity for timely intervention.

CKD is widely recognized as being closely linked with chronic inflammation and immune system dysfunction. Factors such as elevated pro-inflammatory cytokines, oxidative stress, chronic infections, and gut dysbiosis contribute to immune dysfunction. However, the pathophysiological mechanisms underlying the progression of CKD and the development of associated cardiovascular disease (CVD) are intricate and not fully comprehended. Sterile inflammation, driven by various mediators, such as lipoproteins, uremic toxins, and factors released by damaged cells, plays a significant role in the pathogenesis of progressive CKD and its cardiovascular complications [15]. In the context of sterile inflammation, inflammatory cells increase the secretion of IL-1 and interleukin 6 (IL-6), triggering the release of pro-inflammatory cytokines that are closely linked to the development and progression of CKD and CVD [16]. Several studies have reported elevated levels of inflammatory markers, including C-reactive protein (CRP), tumor necrosis factor alpha (TNF-α), and IL-6, in CKD patients compared to healthy controls [5]. These heightened markers have been shown to have predictive value for mortality risk. Changhyun Lee et al. Demonstrated that, although the predictive performance of hs-CRP was not superior to traditional risk factors, elevated hs-CRP levels were associated with a higher risk of CVD and mortality in Korean CKD patients [17]. Plasma IL-6 was independently shown to predict overall and cardiovascular mortality in patients across different stages of chronic kidney disease [18]. Additionally, Carmine Zoccali et al. found that in CKD patients undergoing dialysis, plasma IL-6 levels were a better predictor of death than interleukin (IL)-1β, tumor necrosis factor (TNF)-α, and CRP levels [19]. In our study, we found that WBC and NEU counts were elevated in patients with high NPAR levels. This finding revealed the presence of enhanced inflammatory cells and an inflammatory state in CKD stages G3a to G5, suggesting that NPAR can predict the inflammatory status in CKD. We confirmed that an elevated NEU count is a predictor of all-cause mortality in patients with CKD stages G3a to G5. However, NPAR has shown a superior ability to predict both all-cause mortality and cardiovascular mortality compared to NEU alone.

Recently, the nephrology community has shown growing interest in studies that focusing on nutritional interventions. Increasing evidence suggests that nutritional intervention strategies can ameliorate renal injury in both acute and chronic scenarios through diverse immunomodulatory mechanisms. Ana Cardoso et al. found that hypoalbuminaemia is an independent risk factor for early mortality in hemodialysis (HD) patients with heart failure (HF), patients with hypoalbuminaemia also had higher levels of inflammatory markers, such as serum ferritin and CRP [20]. Additionally, a 10-year cohort study from Japan found that HD patients with serum albumin levels >3.8 g/dL had better survival rates [21]. The supplementation of fish oil in the human diet has been shown to reduce the production of thromboxane B2, 5-hydroxyeicosatetraenoic acid, leukotriene E4, TNF-α, and IL-1β by inflammatory cells such as neutrophils and macrophages [22]. Fish oil has also been found to reduce levels of CRP, IL-6, and IL-1β [23]. The NF-κB signaling pathway is a classic pathway for inducing inflammation, and is involved in the pathogenesis of renal inflammation caused by infection, injury, or autoimmune factors [24]. S. S. Ghosh et al. demonstrated that curcumin dose-dependently antagonized TNF-α-mediated reductions in PPARγ and blocked the transactivation of NF-κB and repression of PPARγ, indicating that curcumin supplementation may ameliorate inflammation and chronic renal failure (CRF) [25]. Therefore, accurately assessing the inflammatory and nutritional status in patients with CKD is critical for identifying the optimal timing for nutritional supplementation. In our study, we did not observe that ALB served as a predictor for all-cause and CVD mortality in patients with CKD stages G3a to G5. However, as a composite indicator combining NEU and ALB, NPAR exhibited a robust predictive capacity in this context. NPAR, as a novel inflammatory-nutritional marker, can accurately assess both inflammation and nutritional status in patients with CKD stages G3a to G5.

The inflammatory markers mentioned previously, such as TNF-α and IL-1β, present challenges in clinical measurement and are considered non-routine laboratory tests. Consequently, more accessible and cost-effective composite inflammatory indices have been identified as effective tools for evaluating inflammation and nutritional status in patients with CKD. Several studies have investigated the association between composite inflammatory indices and all-cause or CVD mortality in CKD. Jialing Zhang et al. found that a high NLR value was associated with an increased risk of all-cause mortality, while PLR proved to be a better predictor of CV mortality in maintenance HD patients [26]. However, a study by K.J. Mayne et al. demonstrated that NLR was strongly associated with mortality in hemodialysis patients, while the association between PLR and adverse outcomes was weaker [27]. Wenwu Liu et al. proposed an alternative perspective, suggesting that MLR is more effective than NLR in predicting mortality risk among patients with CKD, based on data analysis from NHANES 2003–2010 [28]. In the study by Jiaxian Liao et al. NLR, MLR and PLR were conducted as an inflammation score, which was shown to have an independent association with all-cause mortality in hemodialysis patients [29]. SIRI, a novel composite index that integrates three independent white blood cell subsets [30], has been found to independently predict the risk of both all-cause and cardiovascular mortality in CKD patients, with potentially greater significance in the early stages (Stage I to Stage III) of CKD, as shown in a cohort study [31]. We compared the predictive capabilities of NPAR with NLR, SIRI, NPHR, and NEU for all-cause and cardiovascular mortality in CKD stages G3a-G5. Our findings revealed that NPAR exhibited superior predictive performance, with notable advantages in predicting mortality risk from diabetes and non-cardiovascular deaths as well.

Our study revealed that both ALB and NEU lacked predictive power for all-cause mortality and were ineffective in predicting cardiovascular mortality risk in CKD patients. Nonetheless, NPAR, calculated as the neutrophil percentage divided by serum albumin concentration, demonstrated superior prognostic value compared to ALB or NEU alone. Initially identified as a biomarker for predicting prognosis in patients with rectal cancer [32] and end-stage pancreatic cancer [33], NPAR has also been reported as an independent risk factor for in-hospital mortality in patients with coronary artery stenosis in the CCU and for 1-year mortality in patients with advanced heart failure [9,34]. The relationship between NPAR and acute kidney injury (AKI) has also been established. Benji Wang et al. found that an increased NPAR correlates with a higher risk of all-cause mortality in critically ill patients with AKI [35]. NPAR has also been identified as an independent predictor of contrast-associated acute kidney injury (CA-AKI) and long-term mortality in patients without CKD undergoing elective percutaneous coronary intervention (PCI) [^,^^12^]. Youqun Gao, BS et al. [36] and Yi Yu et al. [37] demonstrated that elevated NPAR levels were independently associated with an increased risk of all-cause and cardiovascular mortality in peritoneal dialysis (PD) patients. Currently, limited research has explored the relationship between NPAR and CKD. NPAR has been independently associated with the severity of coronary atherosclerosis in CKD patients, especially in female and elderly patients (≥65 years old). Moreover, the NPAR effectively predicts the severity of coronary atherosclerosis, with greater predictive value in females than in males [38]. Jinxi Li et al. [39] provided strong evidence supporting the association between elevated NPAR levels and CKD. To date, no studies have examined the correlation between NPAR and mortality rates in CKD. Our research stands as the first to reveal that NPAR has predictive value for overall mortality, cardiovascular mortality, non-cardiovascular mortality, and diabetes-related mortality among individuals with CKD stages G3a through G5. The adjusted model, which has undergone rigorous internal validation, demonstrates that NPAR is more effective in forecasting CKD mortality risk.

The strengths of our study include the utilization of an extensive national database and a prospective cohort study with a long follow-up period. NHANES employs standardized protocols for data collection, executed by professional and well-trained staff. These protocols involve the use of standard questionnaires, physical examinations, and laboratory analyses. To our knowledge, this is the first study to investigate the correlation between NPAR and both all-cause and specific mortality among individuals with CKD stages G3a-G5. Furthermore, we conducted a model and adjusted for various covariates to assess the prognostic ability for NPAR and performed rigorous internal validation to explore these associations across diverse populations, revealing a non-linear relationship between NPAR and CKD mortality.

Some limitations remain in our study. First, the NHANES surveys rely on individual interviews and questionnaires, which may result in inaccuracies in reporting or recall bias. Secondly, in the selection of participants with CKD stages G3a-G5, certain data were found to be absent. We employed a direct deletion approach, which could have potentially introduced bias. Thirdly, despite the adjusted Cox regression model underwent thorough internal validation, additional external validation would be more effective in demonstrating the predictive capabilities of the model for NPAR and CKD mortality risk.

## Conclusions

In conclusion, this study is the first to identify the NPAR as a novel, potential, and independent prognostic indicator for CKD stages G3a to G5. NPAR can predict both all-cause and cardiovascular disease clinical outcomes in CKD patients. Compared to those with low NPAR levels, individuals with high NPAR levels are at increased risk of mortality and have reduced survival in the context of CKD. This finding provides evidence supporting the timely evaluation and intervention for inflammation and nutritional status in individuals with CKD stages G3a to G5. Additionally, we found that NPAR has predictive value for diabetes-related mortality among CKD G3a to G5 patients, which requires further validation.

## Supplementary Material

Supplemental Material

Supplemental Material

Supplemental Material

Supplemental Material

Supplemental Material

Supplemental Material

## Data Availability

Publicly available datasets were analyzed in this study. This data can be found here: https://www.cdc.gov/nchs/nhanes/index.htm.
