# Supplementary material for: The prognostic value of the neutrophil-percentage-to-albumin ratio for all-cause and cardiovascular mortality in chronic kidney disease stages G3a to G5: insights from NHANES 2003–2018
Source: Ren Fail. 2025 May 7;47(1):2495861. doi: 10.1080/0886022X.2025.2495861 (PMC12064118; doi:10.1080/0886022X.2025.2495861)
Supplement: Supplemental Material [file IRNF_A_2495861_SM6032.docx]

| **CVD mortality** | **model0** | | | **model1** | |  | **model2** | | |  | **model3** | | |
| --- | --- | --- | --- | --- | --- | --- | --- | --- | --- | --- | --- | --- | --- |
| **Variables** | **Crude**  **HR(95% CI)** | **Crude**  **P** |  | **Adjusted**  **HR(95% CI)** | **Adjusted**  **P** |  | **Adjusted**  **HR(95% CI)** | **Adjusted**  **P** | |  | **Adjusted**  **HR(95% CI)** | **Adjusted**  **P** | |
| **Overall patients** |  |  |  |  |  |  |  | |  |  |  | |  |
| **As continuous (per SD)** | 1.135(1.1- 1.17) | **<0.0001** |  | 1.153(1.115-1.193) | **<0.0001** |  | 1.202(1.123-1.287) | | **<0.0001** |  | 1.181(1.1-1.268) | | **<0.0001** |
| **By NPAR cut-off** |  |  |  |  |  |  |  | |  |  |  | |  |
| NPAR<14.512 | 1 |  |  | 1 |  |  | 1 | |  |  | 1 | |  |
| NPAR≥14.512 | 2.105(1.714-2.585) | **<0.0001** |  | 1.949(1.583-2.398) | **<0.0001** |  | 1.593(1.198-2.118) | | **0.001** |  | 1.513(1.131-2.024) | | **0.005** |
| **By NAPR cut-off** |  |  |  |  |  |  |  | |  |  |  | |  |
| NPAR≥14.512 | 1 |  |  | 1 |  |  | 1 | |  |  | 1 | |  |
| NPAR<14.512 | 0.475(0.387-0.583) | **<0.0001** |  | 0.513(0.417-0.632) | **<0.0001** |  | 0.628(0.472-0.834) | | **0.001** |  | 0.661(0.494-0.884) | | **0.005** |
| **Non-CVD mortality** | **model0** | | | **model1** | | | **model2** | | | | **model3** | | |
| **Variables** | **Crude**  **HR(95% CI)** | **Crude**  **P** |  | **Adjusted**  **HR(95% CI)** | **Adjusted**  **P** |  | **Adjusted**  **HR(95% CI)** | **Adjusted**  **P** | |  | **Adjusted**  **HR(95% CI)** | | **Adjusted**  **P** |
| **Overall patients** |  |  |  |  |  |  |  | |  |  |  | |  |
| **As continuous (per SD)** | 1.12（1.095-1.145） | **<0.0001** |  | 1.131（1.104-1.159） | **<0.0001** |  | 1.178（1.122-1.238） | | **<0.0001** |  | 1.18（1.123-1.243） | | **<0.0001** |
| **By NPAR cut-off** |  |  |  |  |  |  |  | |  |  |  | |  |
| NPAR<14.512 | 1 |  |  | 1 |  |  | 1 | |  |  | 1 | |  |
| NPAR≥14.512 | 1.642（1.429-1.888） | **<0.0001** |  | 1.504（1.307-1.732） | **<0.0001** |  | 1.144（0.941-1.392） | | 0.177 |  | 1.16（0.951-1.423） | | 0.14 |
| **By NAPR cut-off** |  |  |  |  |  |  |  | |  |  |  | |  |
| NPAR≥14.512 | 1 |  |  | 1 |  |  | 1 | |  |  | 1 | |  |
| NPAR<14.512 | 0.609（0.53-0.7） | **<0.0001** |  | 0.665（0.577-0.765） | **<0.0001** |  | 0.873（0.719-1.063） | | 0.177 |  | 0.86（0.703-1.051） | | 0.14 |

Supplement Table 2. Cox models of NPAR for CVD mortality and non-CVD mortality in CKD stage G3a-5D participants.

Abbreviation: CVD, cardiovascular; CKD, chronic kidney disease; HR, Hazard Ratio; CI, Confidence Interval; SD, standard deviation; NPAR, neutrophil percentage-to-albumin ratio.
