# Supplementary material for: The prognostic value of the neutrophil-percentage-to-albumin ratio for all-cause and cardiovascular mortality in chronic kidney disease stages G3a to G5: insights from NHANES 2003–2018
Source: Ren Fail. 2025 May 7;47(1):2495861. doi: 10.1080/0886022X.2025.2495861 (PMC12064118; doi:10.1080/0886022X.2025.2495861)
Supplement: Supplemental Material [file IRNF_A_2495861_SM6031.docx]

| **Variables** | **AUC** | **95% CI** | **Cut-off value** | **Sensitivity** | **Specifcity** | **Youden’s index** |
| --- | --- | --- | --- | --- | --- | --- |
| **All-cause mortality** | | | | | | |
| NPAR | 0.601 | 0.581–0.622 | 14.512 | 0.601 | 0.436 | 0.165 |
| NLR | 0.6 | 0.58-0.621 | 2.774 | 0.436 | 0.284 | 0.152 |
| NPHR | 0.588 | 0.568-0.609 | 4.4 | 0.61 | 0.473 | 0.137 |
| SIRI | 0.592 | 0.572-0.612 | 1.323 | 0.595 | 0.435 | 0.16 |
| NEU | 0.551 | 0.53–0.571 | 5.05 | 0.345 | 0.255 | 0.09 |
| ALB | 0.504 | 0.483–0.525 | 4.25 | 0.366 | 0.314 | 0.022 |
| **Diabetes mortality** | | | | | | |
| **Variables** | **AUC** | **95% CI** | **Cut-off value** | **Sensitivity** | **Specifcity** | **Youden’s index** |
| NPAR | 0.631 | 0.584-0.678 | 17.418 | 0.377 | 0.154 | 0.223 |
| NLR | 0.594 | 0.546-0.641 | 2.26 | 0.689 | 0.503 | 0.186 |
| NPHR | 0.621 | 0.576-0.666 | 4.96 | 0.497 | 0.291 | 0.206 |
| SIRI | 0.572 | 0.525-0.618 | 1.61 | 0.527 | 0.366 | 0.161 |
| NEU | 0.575 | 0.526-0.623 | 5.45 | 0.371 | 0.213 | 0.158 |
| ALB | 0.544 | 0.499-0.589 | 4.15 | 0.527 | 0.441 | 0.086 |

Table S1. Multiple ROC in all-cause and diabetes mortality mortality.

Abbreviation: AUC,area under the curve；CI, Confidence Interval; NPAR, neutrophil percentage-to-albumin ratio; NLR, neutrophil-to-lymphocyte ratio; NPHR, neutrophil-to-hemoglobin ratio; SIRI, systemic inflammation response index; NEU, neutrophil; ALB, serum albumin.
