# Supplementary material for: The prognostic value of the neutrophil-percentage-to-albumin ratio for all-cause and cardiovascular mortality in chronic kidney disease stages G3a to G5: insights from NHANES 2003–2018
Source: Ren Fail. 2025 May 7;47(1):2495861. doi: 10.1080/0886022X.2025.2495861 (PMC12064118; doi:10.1080/0886022X.2025.2495861)
Supplement: Supplemental Material [file IRNF_A_2495861_SM6030.docx]

| **Characteristic** | **Overall**  **participants**  **(n = 3,331)** | **NPAR**  **<14.512**  **(n = 1,456)** | **NPAR**  **≥14.512**  **(n = 1,875)** | **SMD** |
| --- | --- | --- | --- | --- |
| **Age**, Mean (SD) | 72.45 (10.26) | 71.65 (10.06) | 73.08 (10.36) | 0.140 |
| **Gender**, n (%) |  |  |  | **0.011** |
| Male | 1,593 (48%) | 692 (48%) | 901 (48%) |  |
| Female | 1,738 (52%) | 764 (52%) | 974 (52%) |  |
| **Race**, n (%) |  |  |  | 0.146 |
| Mexican American | 302 (9.6%) | 111 (8.1%) | 191 (11%) |  |
| Other Hispanic | 165 (5.2%) | 86 (6.3%) | 79 (4.4%) |  |
| Non-Hispanic White | 2,064 (65%) | 842 (62%) | 1,222 (68%) |  |
| Non-Hispanic Black | 623 (20%) | 328 (23%) | 295 (17%) |  |
| Other Race | 177(0.2%) | 89 (0.6%) | 88(0.5%) |  |
| **AFI**, Mean (SD) | 9.57 (15.19) | 9.81 (15.49) | 9.37 (14.96) | **0.029** |
| **BMI**, Mean (SD) | 29.62 (6.71) | 29.27 (6.03) | 29.90 (7.20) | **0.095** |
| **Waist Circ(cm)**, Mean (SD) | 103.60 (14.99) | 102.62 (14.11) | 104.42 (15.64) | 0.121 |
| **Arm Circ (cm)**, Mean (SD) | 32.60 (5.14) | 32.74 (4.90) | 32.50 (5.34) | **0.047** |
| **UACR (mg/g)**, n (%) |  |  |  | 0.344 |
| Normal (<30) | 2,062 (66%) | 1,022 (73%) | 1,040 (60%) |  |
| Moderate (30-300) | 738 (23%) | 296 (21%) | 442 (25%) |  |
| Severe (300-1000) | 179 (5.7%) | 63 (4.5%) | 116 (6.7%) |  |
| Marked (≥1000) | 164 (5.2%) | 25 (1.8%) | 139 (8.0%) |  |
| **eGFR (mL/min/1.73 m²)**, n (%) |  |  |  | 0.280 |
| G3a(45-60) | 2,137 (64%) | 1,043 (72%) | 1,094 (58%) |  |
| G3b(30-44) | 829 (25%) | 303 (21%) | 526 (28%) |  |
| G4(15-29) | 263 (7.9%) | 80 (5.5%) | 183 (9.8%) |  |
| G5(<15) | 102 (3.1%) | 30 (2.1%) | 72 (3.8%) |  |
| **WBC (×10^9^/L)**, Mean (SD) | 7.60 (7.87) | 7.48 (11.39) | 7.70 (3.07) | **0.027** |
| **LYM (×10^9^/L)**, Mean (SD) | 2.17 (6.84) | 2.86 (10.29) | 1.62 (0.58) | 0.170 |
| **MON (×10^9^/L)**, Mean (SD) | 0.62 (0.25) | 0.63 (0.28) | 0.61 (0.21) | 0.068 |
| **NEU (×10^9^/L)**, Mean (SD) | 4.53 (2.25) | 3.68 (1.44) | 5.19 (2.52) | 0.737 |
| **RBC (×10^12^/L)**, Mean (SD) | 4.39 (0.56) | 4.45 (0.53) | 4.34 (0.57) | 0.199 |

Table S3. Baseline characteristics of participants stratified by NPAR evaluated using standardized mean difference.

(continued)

Abbreviation:SMD,standardized mean difference;NPAR,Neutrophil-to-Albumin Ratio; AFI,annual family income; BMI, body mass index; Waist Circ, waist circumference; Arm Circ, arm circumference; UACR, urinary albumin-to-creatinine ratio; eGFR, estimated glomerular filtration rate; WBC,white blood cell count; LYM, lymphocyte number; MON, monocyte number; NEU, neutrophil; RBC, red blood cell; Hb, hemoglobin; PLT, platelet; AST, aspartate aminotransferase; ALP, alkaline phosphatase; ALB, serum albumin; Chol, cholesterol; Ca, calcium; P, phosphorus; UA, uric acid; BUN, blood urea nitrogen; Cr, creatinine; Na, sodium; K, potassium; Cl, chloride; NLR, neutrophil-to-lymphocyte ratio; PLR, platelet-to-lymphocyte ratio; MLR, monocyte-to-lymphocyte ratio; NPHR, neutrophil-to-hemoglobin ratio; SIRI, systemic inflammation response index.

| **Characteristic** | **Overall**  **participants**  **(n = 3,331)** | **NPAR**  **<14.512**  **(n = 1,456)** | **NPAR**  **≥14.512**  **(n = 1,875)** | **SMD** |
| --- | --- | --- | --- | --- |
| **Hb (g/dL)**, Mean (SD) | 13.39 (1.64) | 13.59 (1.51) | 13.23 (1.71) | 0.225 |
| **PLT (×10^9^/L)**, Mean (SD) | 233.47 (73.00) | 231.15 (67.86) | 235.27 (76.73) | **0.057** |
| **AST (U/L)**, Mean (SD) | 24.88 (12.57) | 25.73 (10.91) | 24.23 (13.68) | 0.121 |
| **ALP (U/L)**, Mean (SD) | 76.26 (29.53) | 70.63 (23.64) | 80.62 (32.74) | 0.350 |
| **Chol (mg/dL)**, Mean (SD) | 187.76 (45.44) | 194.37 (45.35) | 182.63 (44.85) | 0.260 |
| **ALB(mg/dL)**,Mean (SD) | 4.06 (0.37) | 4.22 (0.29) | 3.94 (0.37) | 0.828 |
| **Ca (mg/dL)**, Mean (SD) | 9.44 (0.46) | 9.52 (0.41) | 9.37 (0.49) | 0.327 |
| **Iron (mg/dL)**, Mean (SD) | 13.85 (5.20) | 14.67 (4.89) | 13.22 (5.34) | 0.283 |
| **P (mg/dL)**, Mean (SD) | 3.81 (0.66) | 3.82 (0.60) | 3.81 (0.70) | **0.026** |
| **UA(mg/dL)**, Mean (SD) | 6.57 (1.65) | 6.48 (1.53) | 6.64 (1.74) | 0.101 |
| **BUN(umol/L)**, Mean (SD) | 23.71(10.63) | 21.93 (9.32) | 25.09(11.36) | 0.304 |
| **Cr(mg/dL)**, Mean (SD) | 1.56 (1.16) | 1.47 (1.02) | 1.63 (1.26) | 0.138 |
| **Na(mmol/L)**, Mean (SD) | 139.66 (2.84) | 139.69 (2.67) | 139.63 (2.96) | **0.019** |
| **K(mmol/L)**, Mean (SD) | 4.21 (0.46) | 4.16 (0.45) | 4.24 (0.47) | 0.172 |
| **Cl(mmol/L)**, Mean (SD) | 103.15(3.81) | 103.18(3.47) | 103.12(4.06) | **0.016** |
| **NLR**, Mean (SD) | 2.69 (1.63) | 1.66 (0.55) | 3.48 (1.74) | 1.412 |
| **PLR**, Mean (SD) | 137.45(65.32) | 109.02(45.55) | 159.52(69.69) | 0.858 |
| **MLR**, Mean (SD) | 0.36 (0.17) | 0.29 (0.13) | 0.41 (0.18) | 0.818 |
| **NPHR**, Mean (SD) | 4.59 (1.00) | 3.90 (0.70) | 5.13 (0.86) | 1.576 |
| **SIRI**, Mean (SD) | 1.67 (1.27) | 1.04 (0.56) | 2.17 (1.44) | 1.024 |
| **Hypertension**,n (%) | 2,419 (73%) | 1,053 (72%) | 1,366 (73%) | **0.012** |
| **Diabetes**, n (%) | 1,010 (30%) | 368 (25%) | 642 (34%) | 0.197 |
| **Dialysis**, n (%) | 90 (2.7%) | 26 (1.8%) | 64 (3.4%) | 0.102 |
