# Supplementary material for: The prognostic value of the neutrophil-percentage-to-albumin ratio for all-cause and cardiovascular mortality in chronic kidney disease stages G3a to G5: insights from NHANES 2003–2018
Source: Ren Fail. 2025 May 7;47(1):2495861. doi: 10.1080/0886022X.2025.2495861 (PMC12064118; doi:10.1080/0886022X.2025.2495861)
Supplement: Supplemental Material [file IRNF_A_2495861_SM6021.docx]

| **Propensity Score Matched Participants** | | | | |
| --- | --- | --- | --- | --- |
| **Characteristic** | **NPAR**  **<14.512**  **(n = 926)** | **NPAR**  **≥14.512**  **(n = 926)** | **Absolute**  **SMD** | ***P*-value** |
| **Age**, Mean (SD) | 73.03 (9.07) | 72.68 (10.40) | 0.0337 | 0.440 |
| **Gender**, n (%) |  |  | 0.0130 | 0.608 |
| Male | 500 (54%) | 488 (52.7%) |  |  |
| Female | 764 (52%) | 974 (52%) |  |  |
| **Race**, n (%) |  |  | 0.0000 | 0.738 |
| Mexican American | 77(8.3%) | 77 (8.3%) |  |  |
| Other Hispanic | 42 (4.5%) | 44 (4.8%) |  |  |
| Non-Hispanic White | 605 (65.3%) | 580 (62.6%) |  |  |
| Non-Hispanic Black | 150 (16.2%) | 171 (18.5%) |  |  |
| Other Race | 52 (5.6%) | 54(5.8%) |  |  |
| **BMI**, Mean (SD) | 29.36 (6.0) | 29.38 (6.6) | 0.0032 | 0.939 |
| **UACR (mg/g)**, n (%) |  |  | 0.0022 | 0.939 |
| Normal (<30) | 647 (69.9%) | 645 (69.7%) |  |  |
| Moderate (30-300) | 212 (22.9%) | 215 (23.2%) |  |  |
| Severe (300-1000) | 46 (5.0%) | 42 (4.5%) |  |  |
| Marked (≥1000) | 21 (2.3%) | 24 (2.6%) |  |  |
| **eGFR (mL/min/1.73 m²)**, n (%) |  |  | 0.0065 | 0.938 |
| G3a(45-60) | 637 (68.8%) | 631 (68.1%) |  |  |
| G3b(30-44) | 218 (23.5%) | 221 (23.9%) |  |  |
| G4(15-29) | 60 (6.5%) | 60 (6.5%) |  |  |
| G5(<15) | 11 (1.2%) | 14 (1.5%) |  |  |
| **WBC (×10^9^/L)**, Mean (SD) | 7.31 (5.20) | 7.46 (2.03) | 0.0489 | 0.403 |
| **RBC (×10^12^/L)**, Mean (SD) | 4.42 (0.53) | 4.42 (0.54) | 0.0123 | 0.778 |
| **Hb (g/dL)**, Mean (SD) | 13.56 (1.51) | 13.55 (1.67) | 0.0047 | 0.914 |
| **PLT (×10^9^/L)**, Mean (SD) | 228.04 (65.67) | 228.36 (66.90) | 0.0043 | 0.916 |
| **AST (U/L)**, Mean (SD) | 24.88 (9.26) | 24.75 (10.93) | 0.0094 | 0.781 |
| **ALP (U/L)**, Mean (SD) | 72.79 (24.31) | 73.35 (24.71) | 0.0173 | 0.625 |
| **Chol (mg/dL)**, Mean (SD) | 189.42 (43.65) | 188.19 (44.15) | 0.0273 | 0.548 |
| **ALB(mg/dL)**,Mean (SD) | 4.13 (0.27) | 4.12 (0.29) | 0.0375 | 0.300 |
| **Ca (mg/dL)**, Mean (SD) | 9.47 (0.39) | 9.47 (0.48) | 0.0040 | 0.924 |
| **Iron (mg/dL)**, Mean (SD) | 14.23 (4.49) | 14.49 (5.62) | 0.0488 | 0.266 |
| **P (mg/dL)**, Mean (SD) | 3.78 (0.57) | 3.78 (0.61) | 0.0005 | 0.991 |
| **UA(mg/dL)**, Mean (SD) | 6.52 (1.52) | 6.49 (1.58) | 0.0134 | 0.748 |
| **BUN(umol/L)**, Mean (SD) | 22.39 (9.24) | 22.65 (9.08) | 0.0240 | 0.531 |
| **Cr(mg/dL)**, Mean (SD) | 1.40 (0.67) | 1.43 (0.70) | 0.0310 | 0.375 |
| **Na(mmol/L)**, Mean (SD) | 139.67 (2.67) | 139.67 (2.86) | 0.0004 | 0.993 |

Table S4. Baseline characteristics of participants stratified by NPAR after propensity score matching.

(continued)

Abbreviation:SMD,standardized mean difference; NPAR, Neutrophil-to-Albumin Ratio; BMI, body mass index; UACR, urinary albumin-to-creatinine ratio; eGFR, estimated glomerular filtration rate; WBC,white blood cell count; RBC, red blood cell; Hb, hemoglobin; PLT, platelet; AST, aspartate aminotransferase; ALP, alkaline phosphatase; ALB, serum albumin; Chol, cholesterol; Ca, calcium; P, phosphorus; UA, uric acid; BUN, blood urea nitrogen; Cr, creatinine; Na, sodium; K, potassium; Cl, chloride.

| **Characteristic** | **NPAR**  **<14.512**  **(n = 1,456)** | **NPAR**  **≥14.512**  **(n = 1,875)** | **Absolute**  **SMD** | ***P*-value** |
| --- | --- | --- | --- | --- |
| **K(mmol/L)**, Mean (SD) | 4.18 (0.45) | 4.19 (0.44) | 0.0353 | 0.428 |
| **Cl(mmol/L)**, Mean (SD) | 103.25 (3.43) | 103.34(3.92) | 0.0212 | 0.622 |
| **Hypertension**,n (%) | 676 (73%) | 672 (72.6%) | 0.0043 | 0.876 |
| **Diabetes**, n (%) | 249 (26.9%) | 263 (28.4%) | 0.0151 | 0.499 |
| **Dialysis**, n (%) | 10 (1.1%) | 9 (1.0%) | 0.0011 | 1.000 |
