# Supplementary material for: The prognostic value of the neutrophil-percentage-to-albumin ratio for all-cause and cardiovascular mortality in chronic kidney disease stages G3a to G5: insights from NHANES 2003–2018
Source: Ren Fail. 2025 May 7;47(1):2495861. doi: 10.1080/0886022X.2025.2495861 (PMC12064118; doi:10.1080/0886022X.2025.2495861)
Supplement: Supplemental Material [file IRNF_A_2495861_SM6014.docx]

Table S5. Cox models of NPAR for all-cause mortality and CVD mortality in CKD stage G3a-5D participants after propensity score matching .

| **Model 0** | | | **Model 1** | | **Model 2** | | **Model 3** | | **Model 4** | | |
| --- | --- | --- | --- | --- | --- | --- | --- | --- | --- | --- | --- |
| **Variables** | **Crude HR**  **(95% CI)** | **Crude**  **P** | **Adjusted HR**  **(95% CI)** | **Adjusted**  ***P*** | **Adjusted HR**  **(95% CI)** | **Adjusted**  ***P*** | **Adjusted HR**  **(95% CI)** | **Adjusted**  ***P*** | **Adjusted HR**  **(95% CI)** | **Adjusted**  ***P*** | |
| **All-cause mortality** | | | | | | | | | | | |
| **As continuous (per SD)** | 1.046 (1.016-1.077) | **0.003** | 1.179 (1.094- 1.271) | **<0.001** | 1.179 (1.094-1.271) | **<0.001** | 1.161 (1.075-1.254) | **<0.001** | 1.161 (1.075-1.254) | | **<0.001** |
| **By NPAR cut-off** | | | | | | | | | | | |
| NPAR<14.512 | 1 |  | 1 |  | 1 |  | 1 |  | **1** | |  |
| NPAR≥14.512 | 1.237 (1.076-1.422) | **0.003** | 1.238 ( 1.077-1.423) | **0.003** | 1.396 (1.133-1.721) | **0.002** | 1.248(1.006-1.547) | **0.044** | 1.267 (1.023-1.570) | | **0.03** |
| **By NPAR cut-off** | | | | | | | | | | | |
| NPAR≥14.512 | 1 |  | 1 |  | 1 |  | 1 |  | 1 | |  |
| NAPR<14.512 | 0.809 (0.703-0.929) | **0.003** | 0.808 (0.703-0.929) | **0.003** | 0.716 (0.581-0.883) | **0.002** | 0.802(0.647-0.994) | **0.044** | 0.789 (0.637-0.977) | | **0.03** |
| **CVD mortality** | | | | | | | | | | | |
| **As continuous (per SD)** | 1.084 (1.029-1.142) | **0.002** | 1.057 (1.026-1.088) | **<0.001** | 1.142 (1.001-1.302) | **0.05** | 1.146 (1.008-1.303) | **0.04** | 1.144 (1.006-1.300) | | **0.04** |
| **By NPAR cut-off** | | | | | | | | | | | |
| NPAR<14.512 | 1 |  | 1 |  | 1 |  | 1 |  | 1 | |  |
| NPAR≥14.512 | 1.668 (1.294-2.151) | **<0.001** | 1.669 (1.294-2.153) | **<0.001** | 1.908(1.299-2.803) | **<0.001** | 1.702 (1.149-2.521) | **0.008** | 1.686 (1.137-2.498) | | **0.009** |
| **By NPAR cut-off** | | | | | | | | | | | |
| NPAR≥14.512 | 1 |  | 1 |  | 1 |  | 1 |  | 1 | |  |
| NAPR<14.512 | 0.599 (0.465-0.773) | **<0.001** | 0.599 (0.464-0.773) | **<0.001** | 0.524 (0.357-0.770) | **<0.001** | 0.588 (0.367-0.870) | **0.008** | 0.593 (0.400-0.879) | | **0.009** |

**Abbreviation:** HR, hazard ratios; CIs, confidence intervals; NPAR, neutrophil percentage-to-albumin ratio; eGFR,estimated glomerular filtration rate; CVD,cardiovascular

disease; CKD,chronic kidney diease. Model 0:unadjusted. Model 1, adjusted for age, sex, and race. Model 2, adjusted for age, sex, race, NEU, Hb, PLT, ALP, BUN, Ca, UA,

K, Cl, NPHR, and UACR. Model 3, adjusted for age, sex, race, BMI, UACR, MON, NEU, RBC, Hb, PLT, NLR, PLR, MLR, NPHR, SIRI, ALP, BUN, Ca, Chol, iron, UA,

Cr, Na, K, Cl, diabetes, and dialysis. Model 4, adjusted the variables in Model 3 + eGFR.
