# Supplementary material for: The prognostic value of the neutrophil-percentage-to-albumin ratio for all-cause and cardiovascular mortality in chronic kidney disease stages G3a to G5: insights from NHANES 2003–2018
Source: Ren Fail. 2025 May 7;47(1):2495861. doi: 10.1080/0886022X.2025.2495861 (PMC12064118; doi:10.1080/0886022X.2025.2495861)
Supplement: Supplemental Material [file IRNF_A_2495861_SM6007.docx]

Table S6.Cox models adjusted for eGFR.

| **Model 4** | | | | |
| --- | --- | --- | --- | --- |
| **Variables** | **Crude HR**  **(95% CI)** | **Crude**  **P** | **Crude HR**  **(95% CI)** | **Crude**  **P** |
| **All-cause mortality** | | | **CVD mortality** | |
| **Overall patients** |  |  |  |  |
| **As continuous (per SD)** | 1.179 (1.132-1.228) | **<0.001** | 1.186 (1.104-1.274) | **<0.001** |
| **By NPAR cut-off** |  |  |  |  |
| NPAR<14.512 | 1 |  | 1 |  |
| NPAR≥14.512 | 1.235 (1.052-1.449) | **<0.01** | 1.519 (1.134-2.034) | **<0.01** |
| **By NPAR cut-off** |  |  |  |  |
| NPAR≥14.512 | 1 |  | 1 |  |
| NAPR<14.512 | 0.810 (0.690-0.950) | **<0.01** | 0.659 (0.492-0.882) | **<0.01** |

Abbreviation: HR, hazard ratios; CIs, confidence intervals; NPAR, neutrophil percentage-to-albumin ratio; eGFR,estimated glomerular

filtration rate; CVD, cardiovascular disease; CKD,chronic kidney diease. Model 4, adjusted for age, sex, race, BMI, UACR, MON, NEU, RBC, Hb, PLT, NLR, PLR, MLR, NPHR, SIRI, ALP, BUN, Ca, Chol, iron, UA, Cr, Na, K, Cl, diabetes, dialysis and eGFR.

Abbreviation: HR, hazard ratios; CIs, confidence intervals; NPAR, neutrophil percentage-to-albumin ratio; eGFR,estimated glomerular

filtration rate; CKD,chronic kidney diease.

Model 0:unadjusted. Model 1, adjusted for age, sex, and race. Model 2, adjusted for age, sex, race, NEU, Hb, PLT, ALP, BUN, Ca, UA, K,

Cl, NPHR, and UACR. Model 3, adjusted for age, sex, race, BMI, UACR, MON, NEU, RBC, Hb, PLT, NLR, PLR, MLR, NPHR, SIRI,

ALP, BUN, Ca, Chol, iron, UA, Cr, Na, K, Cl, diabetes, and dialysis.
